# Development and validation of a reinforcement learning algorithm to dynamically optimize mechanical ventilation in critical care

**DOI:** 10.1038/s41746-021-00388-6

**Published:** 2021-02-19

**Authors:** Arne Peine, Ahmed Hallawa, Johannes Bickenbach, Guido Dartmann, Lejla Begic Fazlic, Anke Schmeink, Gerd Ascheid, Christoph Thiemermann, Andreas Schuppert, Ryan Kindle, Leo Celi, Gernot Marx, Lukas Martin

**Affiliations:** 1 Department of Intensive Care and Intermediate Care, University Hospital RWTH Aachen, Pauwelsstreet 30, Aachen, Germany; 2 Chair for Integrated Signal Processing Systems, RWTH Aachen University, Kopernikusstreet 16, Aachen, Germany; 3 Environmental Campus Birkenfeld, Trier University of Applied Sciences, Schneidershof, Trier, Germany; 4 Research Area Information Theory and Systematic Design of Communication Systems, RWTH Aachen University, Kopernikusstreet 16, Aachen, Germany; 5 William Harvey Research Institute, Queen Mary University London, Charterhouse Square, London, United Kingdom; 6 Joint Research Center for Computational Biomedicine, RWTH Aachen University, Pauwelsstreet 30, Aachen, Germany; 7 Laboratory for Computational Physiology, Harvard–MIT Division of Health Sciences & Technology, Cambridge, MA USA; 8 Division of Pulmonary, Critical Care and Sleep Medicine, Beth Israel Deaconess Medical Center, Boston, MA USA; 9 Department of Biostatistics Harvard T.H, Chan School of Public Health, Boston, MA USA

**Keywords:** Medical research, Translational research

## Abstract

The aim of this work was to develop and evaluate the reinforcement learning algorithm VentAI, which is able to suggest a dynamically optimized mechanical ventilation regime for critically-ill patients. We built, validated and tested its performance on 11,943 events of volume-controlled mechanical ventilation derived from 61,532 distinct ICU admissions and tested it on an independent, secondary dataset (200,859 ICU stays; 25,086 mechanical ventilation events). A patient “data fingerprint” of 44 features was extracted as multidimensional time series in 4-hour time steps. We used a Markov decision process, including a reward system and a Q-learning approach, to find the optimized settings for positive end-expiratory pressure (PEEP), fraction of inspired oxygen (FiO_2_) and ideal body weight-adjusted tidal volume (Vt). The observed outcome was in-hospital or 90-day mortality. VentAI reached a significantly increased estimated performance return of 83.3 (primary dataset) and 84.1 (secondary dataset) compared to physicians’ standard clinical care (51.1). The number of recommended action changes per mechanically ventilated patient constantly exceeded those of the clinicians. VentAI chose 202.9% more frequently ventilation regimes with lower Vt (5–7.5 mL/kg), but 50.8% less for regimes with higher Vt (7.5–10 mL/kg). VentAI recommended 29.3% more frequently PEEP levels of 5–7 cm H_2_O and 53.6% more frequently PEEP levels of 7–9 cmH_2_O. VentAI avoided high (>55%) FiO_2_ values (59.8% decrease), while preferring the range of 50–55% (140.3% increase). In conclusion, VentAI provides reproducible high performance by dynamically choosing an optimized, individualized ventilation strategy and thus might be of benefit for critically ill patients.

## Introduction

Despite intense efforts in basic and clinical research, an individualized ventilation strategy for critically ill patients remains a major challenge^1^. If not applied adequately, suboptimal ventilator settings can result in ventilator-induced lung injury (VILI), hemodynamic instability and toxic effects of oxygen. Pathophysiologically, VILI is triggered by volutrauma (high tidal volumes), barotrauma (high pressures) and/or atelectrauma (low positive end-expiratory pressure (PEEP) levels), mechanisms that are predominantly described in association with the acute respiratory distress syndrome (ARDS)^2–4^. Established ventilation strategies aim at applying appropriate settings for ideal body weight-adjusted tidal volume (Vt), PEEP and fraction of inspired oxygen (FiO_2_)^2^. In terms of lung protective ventilation, solid evidence exists for limiting Vt to 6 ml/kg ideal body weight and driving pressures to 15 mbar^5,6^. Particular patient groups, especially those with a more pronounced severity of illness, may benefit from an individualized, ultraprotective ventilation regime^7,8^. However, an individualized mechanical ventilation approach remains a challenging task: A multitude of factors, e.g., lab values, vitals, comorbidities, disease progression, and other clinical data must be taken into consideration when choosing a patient’s specific optimal ventilation regime. In addition, an iterative re-evaluation of the optimal mechanical ventilation strategy throughout the course of the treatment is mandatory. In particular, in environments with high data density, such as intensive care units (ICUs) or emergency rooms, the amount of acquired data can result in a complex decision-making process, the outcome of which is strongly influenced by experience and medical knowledge of the attending physician^3^. Enabled by the increase of computational power and availability of high-frequency medical data, new computational approaches have been introduced into the decision making process in medicine: Artificial Intelligence (AI) based on Machine Learning (ML) is increasingly used to capture high complexity patterns in medical data and consequently to predict future events in individual patients (personalized medicine)^9^. Of note, a computational approach using Reinforcement Learning (RL), a specific area of ML, has recently been used to assess vasopressor dosing regimes and volume therapy in septic patients^10^. RL aims to find the optimal policy (e.g. optimal therapeutic strategy) for an agent interacting with an unknown environment by attempting to maximize a cumulative reward^11^. Notably, in RL, rewards can be stochastic. This gives more flexibility to the adopted policy, which is, indeed, needed when developing solutions for complex problems, such as finding the optimal therapeutic (ventilation) strategy in critically ill patients^12^. The application of RL to support the attending physician in finding the optimal mechanical ventilation regime for individual patients has not been investigated. In this study, we developed the VentAI algorithm, a computational model using RL, which is able to dynamically support the physician in choosing the optimal, mechanical ventilation regime. VentAI is built and validated on the Medical Information Mart for Intensive Care III database (MIMIC-III), a large ICU dataset consisting of patient data for 61,532 distinct ICU stays with a total of 11,943 mechanical ventilation (volume-controlled) events^13^. We finally tested the performance of VentAI on two independent datasets, the MIMIC-III and the secondary dataset of eICU Collaborative Research Database v2.0 (eICU). The latter comprises patient data for 200,859 patient distinct ICU stays with a total of 25,086 mechanical ventilation events^14^. Including the three dimensions Vt, PEEP, and FiO_2_, VentAI dynamically develops an optimized mechanical ventilation strategy for the individual patient state.

## Results

### VentAI Performance

**Fig. 1:**
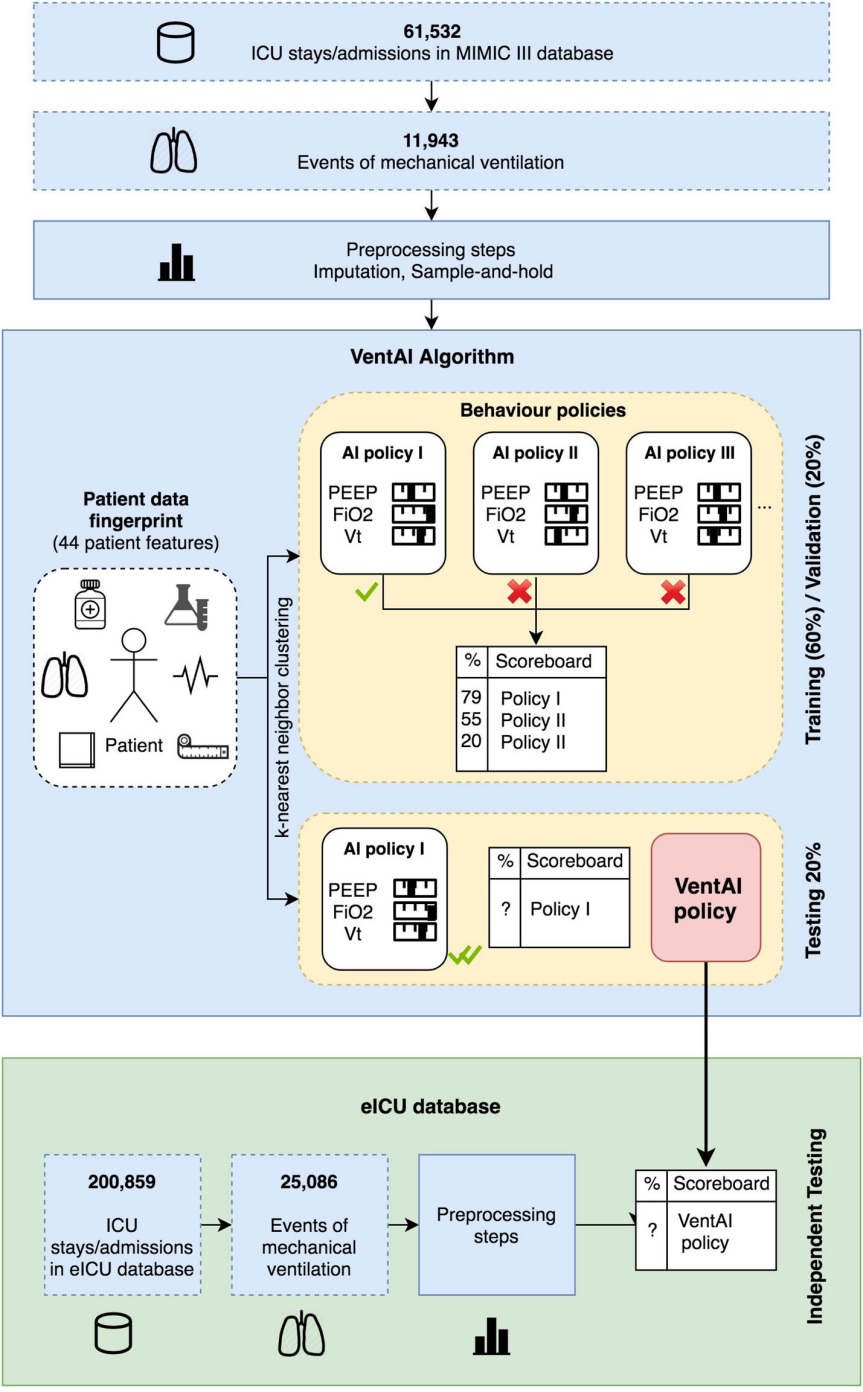
VentAI Data Routine. Flow diagram of the overall cohort, architectural overview of the VentAI algorithm and independent testing on eICU dataset.

The complete dataset from MIMIC-III comprised of 61,532 ICU stays with 11,943 events of mechanical ventilation (Fig. 1). After preprocessing, the cohort (Table 1) has been randomly divided into three datasets (training, validation, and testing) for each model. The evaluation of the performance of the attending physician was conducted via temporal difference Q-learning^15,16^. In order to compare the VentAI (validation and testing) with the clinicians performance, we built a total of 500 models, while the whole learning cycle was repeated for each model.

**Table 1:** Clinical and demographic properties of the study population.

Dataset	MIMIC-III	eICU
Number of ICUs	5	335
Acquisition timespan	2001–2012	2014–2015
Number of included patients	11,443	23,699
Number of mechanical ventilation events	11,943	25,086
Age, years	66.9 (56.3–77.5)	65.0 (54–74)
Body weight, kilogram	85.7 (±18.1)	83.5 (±22.0)
Sex, female	4,329 (36.3%)	10,546 (42.0%)
Sex, male	7,614 (63.7%)	14,540 (58.0)
90-days mortality, %	15.8	Not available
In-hospital mortality, %	11.1	13.2
LOS ICU, days	3.1 (1.6–6.1)	3.0 (1.71–5.9)
LOS hospital, days	9 (6–15)	8 (5–15)
PEEP, cmH_2_O	6 (±1.7)	6.3 (±7.0)
FiO_2_, %	45.9 (±5.8)	44.9 (± 23.6)
Vt, mL/kg	8.3 (±1.6)	7.5 (±1.2)
SOFA at ICU admission, points	5.6 (±2.9)	2.3 (± 2.2)

Demographic and clinical data of the assessed patient cohort, extracted from the MIMIC III and eICU database, respectively. LOS: Length of stay, SOFA: Sequential Organ Failure Assessment Score.

Demographic and clinical data of the assessed patient cohort, extracted from the MIMIC III and eICU database, respectively. Data is presented in *n* (%), mean (SD) or median (IQR); LOS: Length of stay, SOFA: Sequential Organ Failure Assessment Score.

**Fig. 2:**
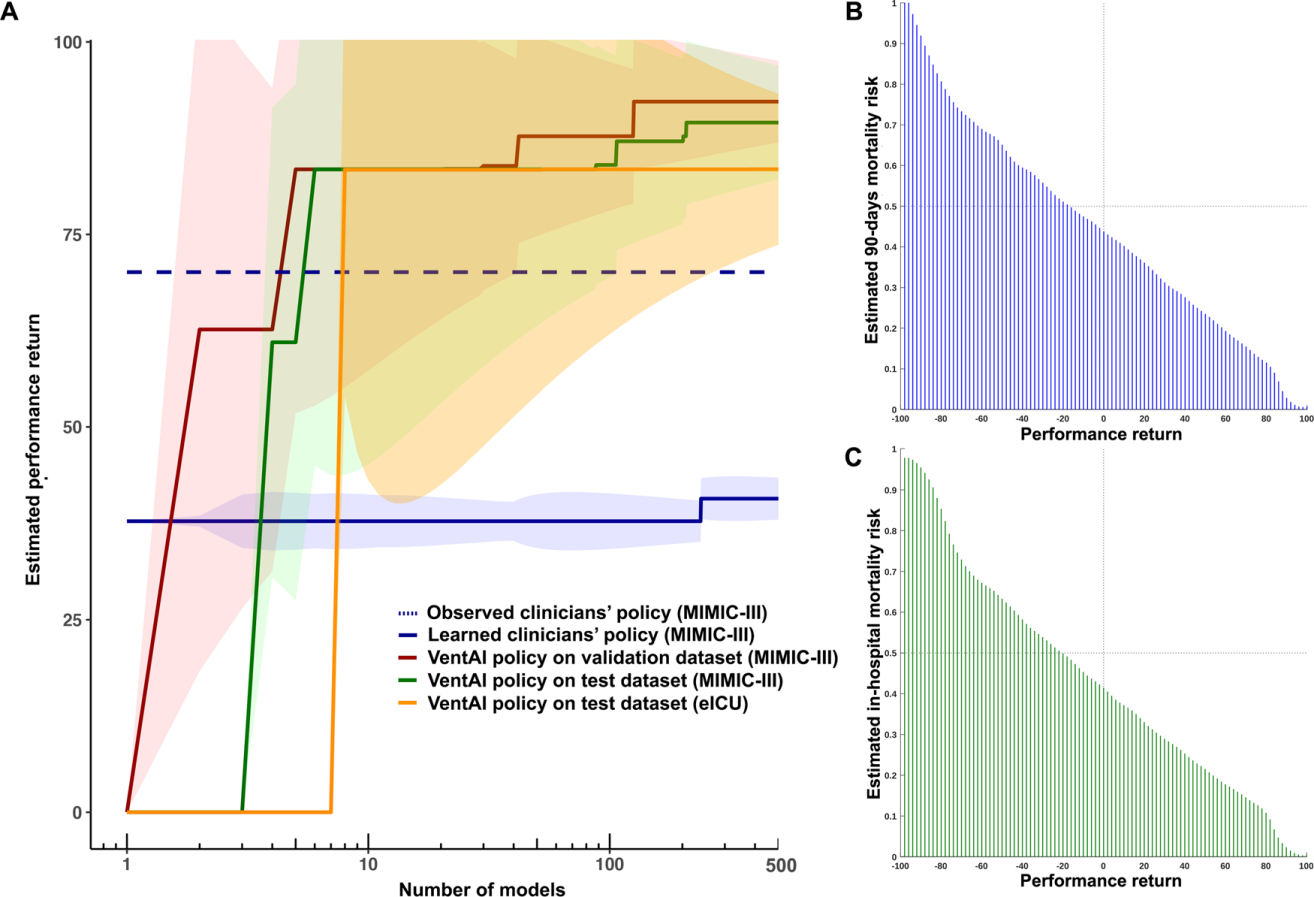
VentAI Performance. **a** VentAI estimated performance return on both datasets (MIMIC-III and eICU) versus clinicians’ performance return with variance in MIMIC-III dataset after the exposure of the policies to 500 models. **b** Relation between VentAI performance return and estimated 90-day mortality risk in the MIMIC-III dataset. **c** Relation between VentAI performance return and in-hospital mortality risk in the eICU dataset.

To evaluate the differences in performance conservatively, we compared the 90% lower bound of the VentAI performance return with the 90% upper bound of the clinicians (Fig. 2), demonstrating the estimated performance return after the exposure of the policies to 500 models. The red line represents the 90% lower bound (LB) for best VentAI policy on MIMIC-III validation data set (2,443 mechanical ventilation events). The green line represents the 90% LB for best VentAI policy on MIMIC-III test data set (2,443 mechanical ventilation events). The orange line represents the 90% LB for best VentAI policy on eICU test data set (25,086 mechanical ventilation events). The estimated clinicians policy performance is shown in blue, representing the 95% upper bound (UB). The shades represent the up-to-the-point cumulative standard deviation across models. VentAI consistently exceeded the clinicians performance return already after four models built. The best dynamically chosen mechanical ventilation regime by the VentAI algorithm resulted in a 93.64 estimated performance return in validation and 91.98 in the testing dataset, respectively. This represents an improvement of 42.6% (40.9% for the test set), compared to the best performance of the clinicians (51.1 estimated performance return), based on the learned model (Fig. 1). In addition, there was an improvement of 22.6% (20.9% for the test set), compared to observable clinician behavior.

### VentAI policy analysis

**Fig. 3:**
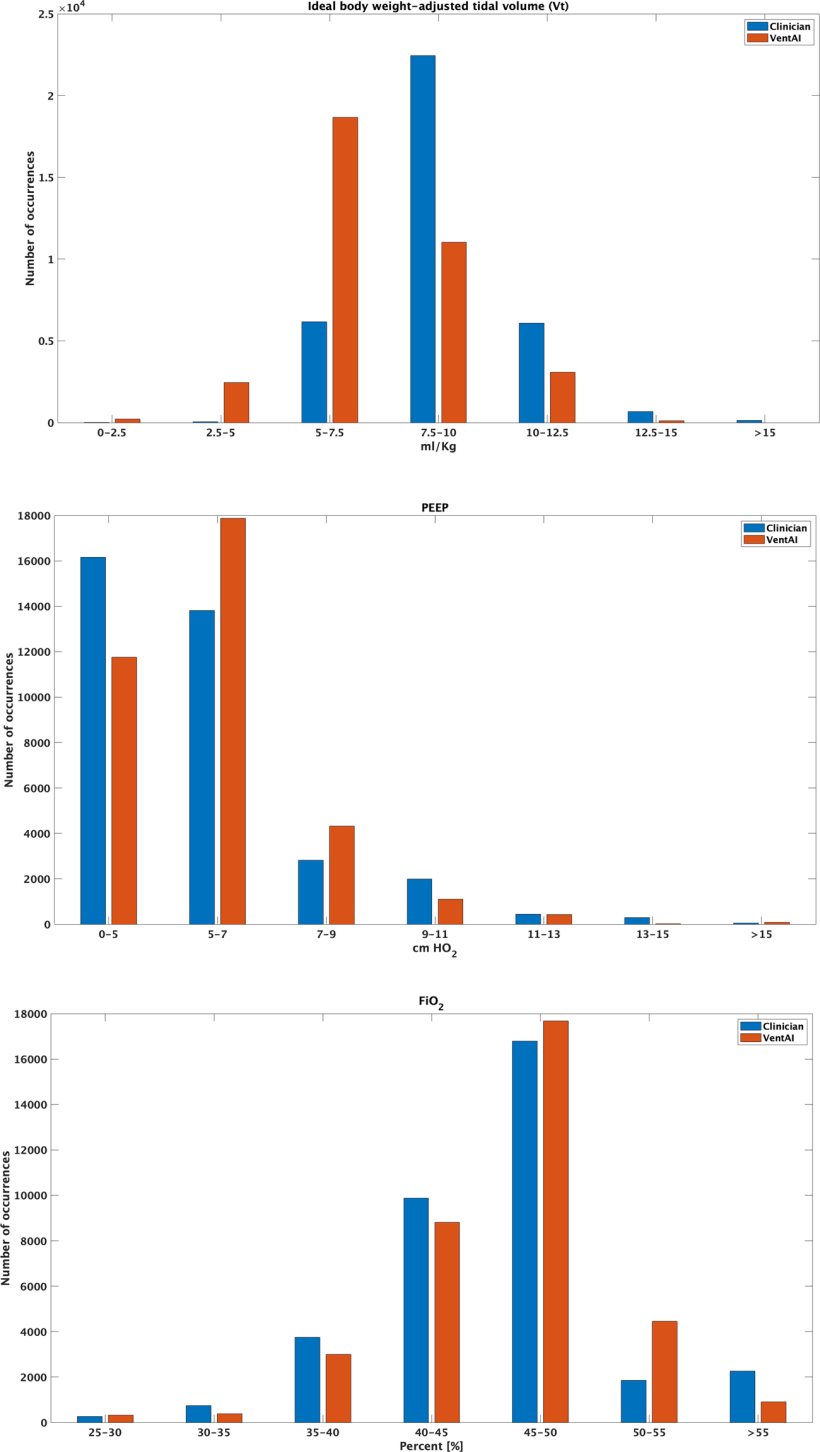
Visualization of the action distribution in the 3-dimensional action space (MIMIC-III dataset). The test set includes 36,225 decision time instances and the designed model facilitates 343 action bins in the action space.

We next elucidated the frequency distributions of the chosen optimal performing VentAI policy, compared to the clinicians, after conducting evaluations on 500 models. We performed a detailed frequency analysis on the three action dimensions (Vt_set_, PEEP, FiO_2_) focusing in particular on the action bins with a change in at least 1% of the total number of possible decision instances (36,225 total decision time instances) (Fig. 3, Tables 2 and 3). This analysis revealed that the VentAI algorithm chose more frequently (202.9% increase relative to the clinicians) ventilation regimes with lower Vtset (5–7.5 mL/kg), but less frequently (50.8% decrease relative to the clinicians) regimes with higher Vt_set_ (7.5–10 mL/kg). Of note, high Vt_set_ settings of >15 ml/kg were avoided completely by VentAI (decrease of 100%). Moreover, VentAI recommended 29.3% more frequently ventilation regimes with PEEP levels of 5–7 cmH_2_O and 53.6% more frequently settings with 7–9 cmH_2_O, compared to the clinicians. Of note, VentAI avoided low PEEP settings of less than 5 cmH_2_O with a relative decrease of 27.3% compared to the clinicians (Fig. 3, Tables 2 and 3, Supplementary Figs. 1–6). Interestingly, the VentAI policy avoided high FiO_2_ values (> 55%) with a decrease of 59.8% relative to clinicians, while preferring FiO_2_ values in the range of 50–55%, indicated by an increase of 140.3% in this range (Fig. 3, Tables 2 and 3).

**Table 2:** Distribution of the chosen action by VentAI in comparison to the clinician’s performance (MIMIC-III dataset).

	1	2	3	4	5	6	7
**Vt (mL/Kg)**	**0–2.5**	**2.5–5**	**5–7.5**	**7.5–10**	**10–12.5**	**12.5–15**	**>15**
*n* (%)	218 (0.6%)	2406 (6.6%)	12504 (34.5%)	−11415 (−31.5%)	−3006 (−8.3%)	−576 (−1.6%)	−131 (−0.36%)
**PEEP (cmH**_**2**_**O)**	**0–5**	**5–7**	**7–9**	**9–11**	**11–13**	**13–15**	**>15**
*n* (%)	−4399 (−12.1%)	4049 (11.1%)	1508 (4.2%)	−895 (−2.4%)	−11 (−0.03%)	−278 (−0.8%)	26 (0.07%)
**FiO**_**2**_ **(%)**	**25–30**	**30–35**	**35–40**	**40–45**	**45–50**	**50–55**	**>55**
*n* (%)	47 (0.12%)	−369 (−1.1%)	−753 (−2.1)	−1069 (−2.9%)	895 (2.4%)	2602 (7.2%)	−1353 (−3.7%)

Ranges for the action space bins are highlighted in bold for Vt, FiO_2_ and PEEP, respectively. Data is presented in total numbers and relative percent of the total numbers of actions chosen (36,225 total decision time instances). Positive numbers indicate that an action has been chosen more frequently by VentAI, negative numbers indicate that an action has been chosen less frequently.

Specific setting ranges for the action space bins are highlighted in grey for Vt, FiO_2_ and PEEP, respectively. Data is presented in total numbers and relative percent of the total numbers of actions chosen (36,225 total decision time instances). Positive numbers indicate that an action has been chosen more frequently by VentAI, negative numbers indicate that an action has been chosen less frequently. Vt: Tidal Volumes; FiO_2_: Fraction of inspired oxygen; PEEP: positive end-expiratory pressure.

**Table 3:** Comparison of percentage of change for each action bin between VentAI policy and clinicians’ policy (MIMIC-III dataset).

	1	2	3	4	5	6	7
**Vt [mL/Kg]**	**0–2.5**	**2.5–5**	**5–7.5**	**7.5–10**	**10–12.5**	**12.5–15**	**>15**
%	*	5119.1	202.9	−50.8	−49.5	−83.7	*
**PEEP [cmH**_**2**_**O]**	**0–5**	**5–7**	**7–9**	**9–11**	**11–13**	**13–15**	**>15**
%	−27.2	29.3	53.6	−44.9	*	*	*
**FiO**_**2**_ **[%]**	**25–30**	**30–35**	**35–40**	**40–45**	**45–50**	**50–55**	**>55**
%	*	*	−20.1	−10.8	5.3	140.3	−59.8

*Less than 1% from the total possible time decision incidence, total table see Table 2c in the Supplemental Files.

Specific setting ranges for the action space bins are highlighted in bold for Vt, FiO_2_ and PEEP, respectively. Data is presented as the percentage of change for each bin by comparing VentAI with the clinician. Positive numbers indicate that an action has been chosen more frequently by VentAI, negative numbers indicate that an action has been chosen less frequently.

Specific setting ranges for the action space bins are highlighted in grey for Vt, FiO_2_ and PEEP, respectively. Data is presented as the percentage of change for each bin by comparing VentAI with the clinician. Positive numbers indicate that an action has been chosen more frequently by VentAI, negative numbers indicate that an action has been chosen less frequently. Vt: Tidal Volumes; FiO_2_: Fraction of inspired oxygen; PEEP: positive end-expiratory pressure.

**Fig. 4:**
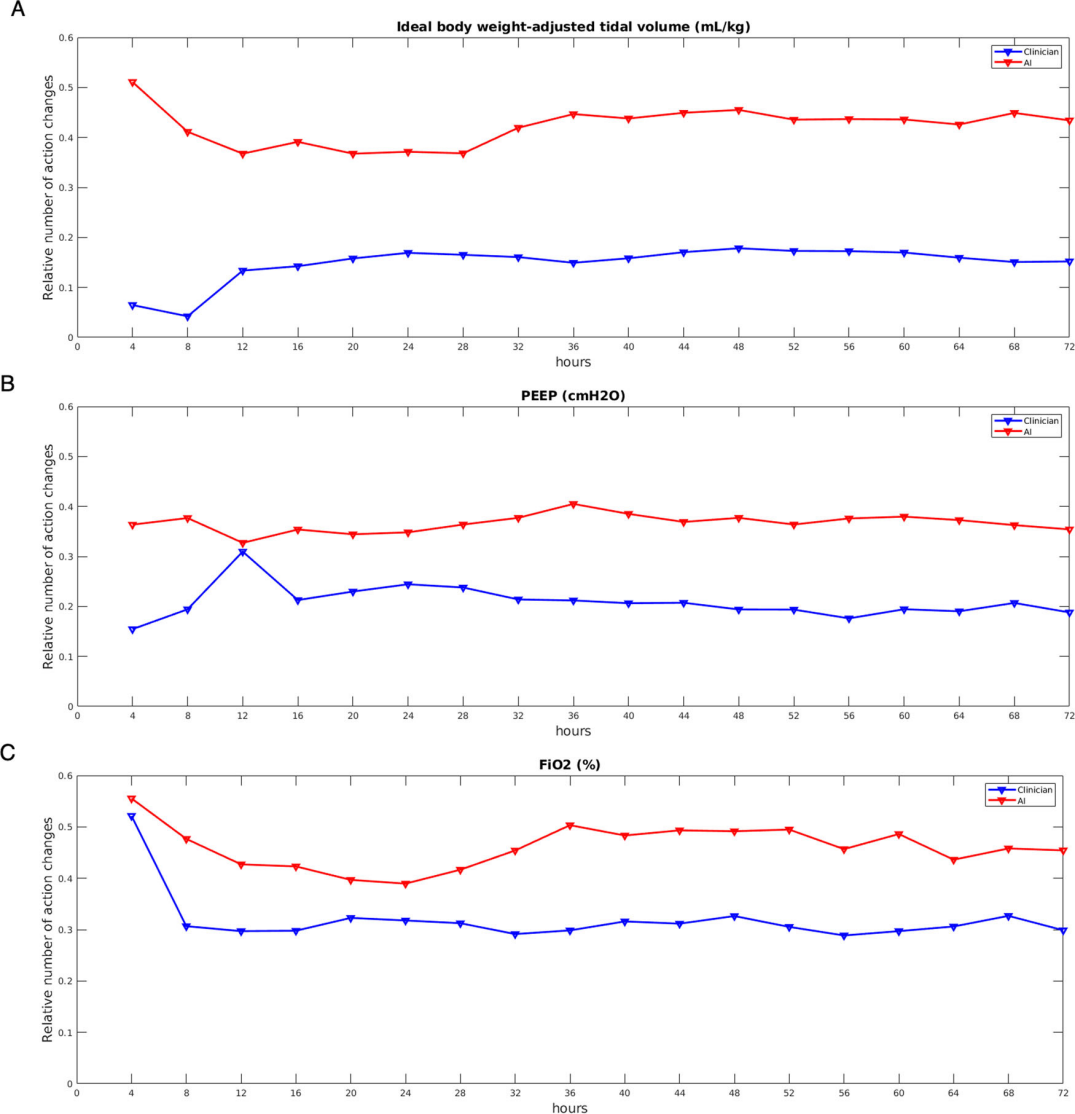
Number of action changes (MIMIC-III dataset). The relative number of action changes (ideal body weight-adjusted tidal volume (Vt), positive end expiratory pressure (PEEP), and fraction of inspired oxygen (FiO_2_)) is shown in relation to the number of mechanically ventilated patients at each 4 h time step. Clinicians action changes are shown in blue while the VentAI action changes are shown in red.

Having shown that VentAI exceeded the clinicians estimated performance and observed policy by adopting Vtset, PEEP, and FiO_2_ with different frequency distributions within the test set, we analyzed the dynamics of VentAI by observing the number of action changes performed at each 4-h time-step during the 72 h observation period (Fig. 4a–c). Of note, the number of action changes per mechanically ventilated patient, chosen by VentAI, were constantly above the number of action changes chosen by the clinicians over the whole observation period (Fig. 4a–c), underlining the high dynamicity of the VentAI algorithm.

**Fig. 5:**
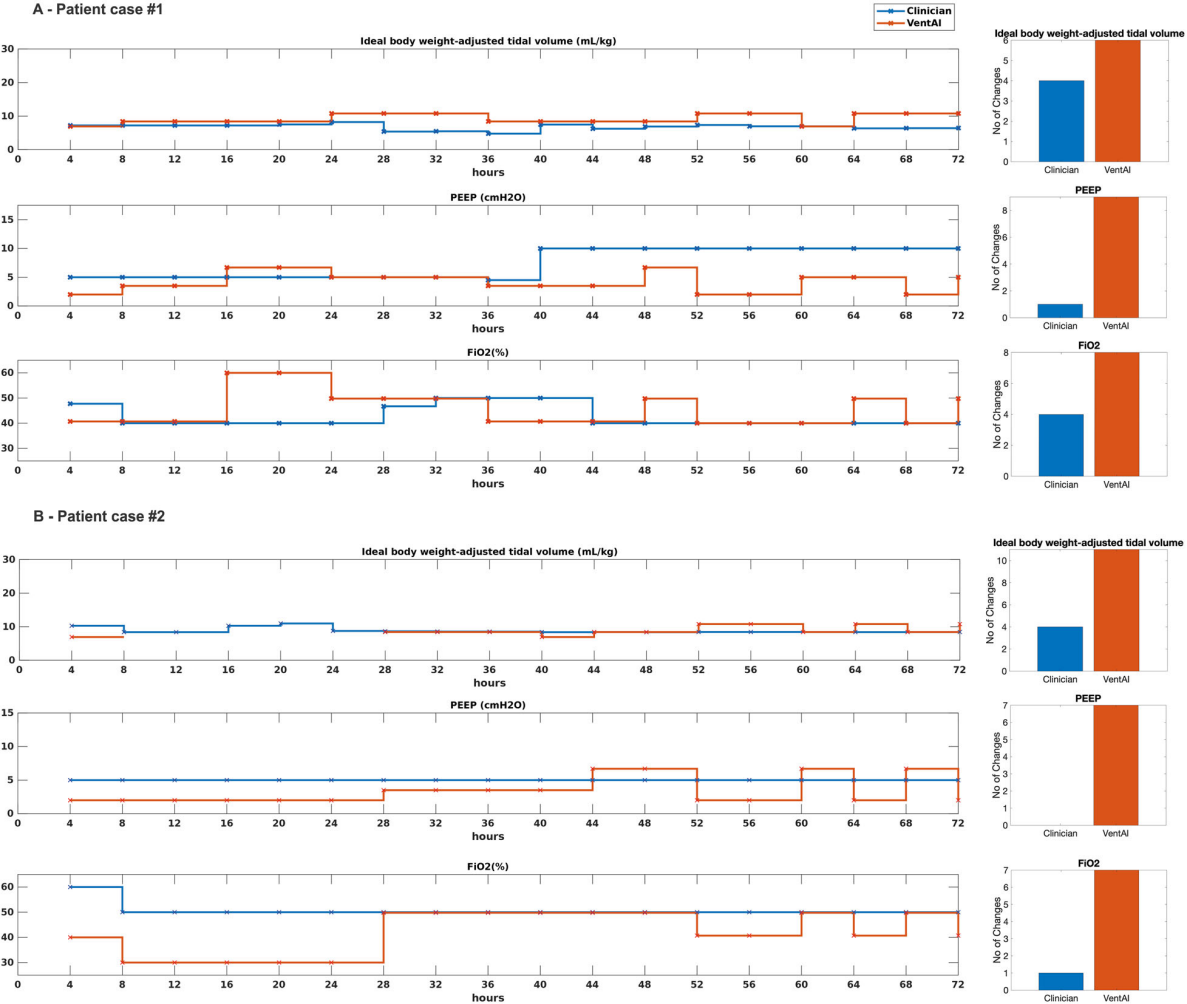
Visualization of two representative patient cases (MIMIC-III dataset). Visualization of two representative case studies in 4-hour intervals. Both patients died within the observed 90 days. Clinicians’ actions are shown in blue while the VentAI actions are shown in red.

**Fig. 6:**
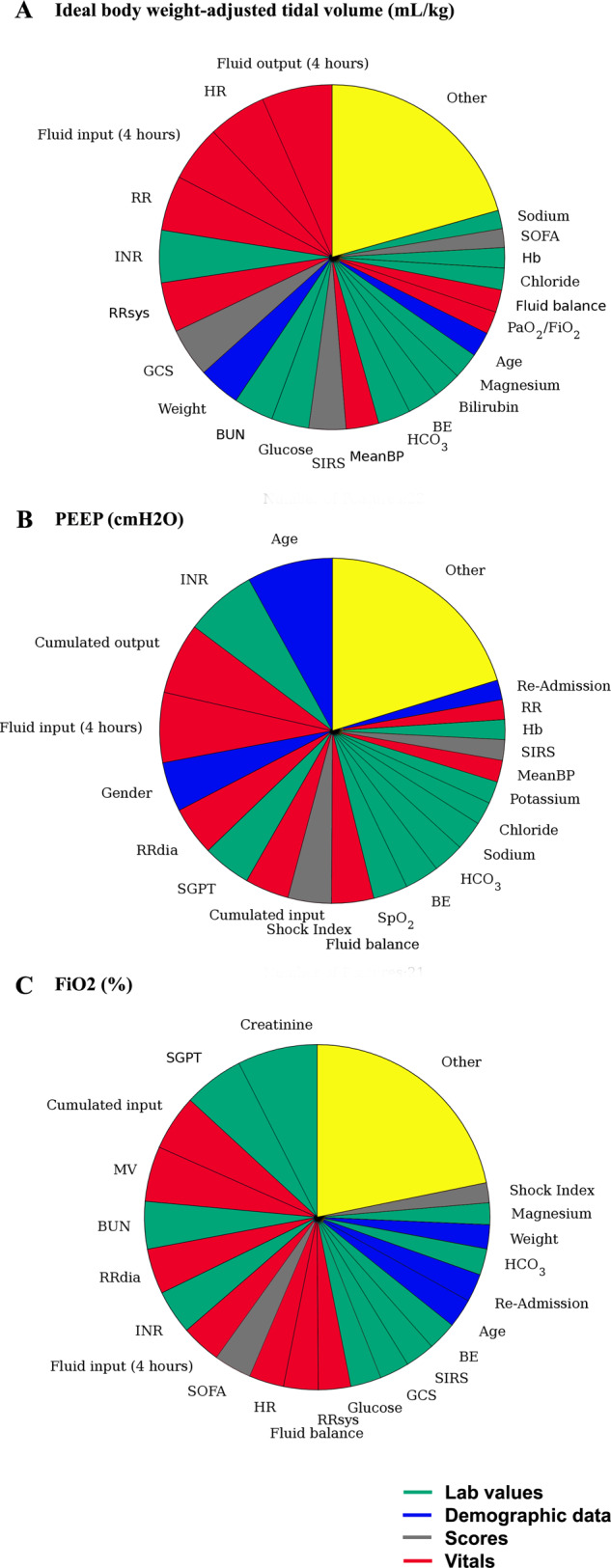
Out-of-Bag feature weight analysis of VentAI (MIMIC-III dataset). Relative weight of each feature using out-of-bag feature weight analysis, based on the relative loss of prediction, represented by an increase of the mean squared error. **a** Ideal body weight-adjusted tidal volume (mL/kg). **b** PEEP (cmH_2_0). **c** FiO_2_ (%).

In order to visualize the dynamic, individualized approach of VentAI, we present two representative individual courses of patient treatment (Fig. 5a, b). Patient #1 was 46 y/o male, admitted as an emergency to the Surgical Intensive Care Unit with end stage renal disease and in need of mechanical ventilation due to respiratory decompensation. Patient #2 was a 82 y/o male, admitted to the cardiac surgery recovery unit in need of mechanical ventilation due to pleural effusions. Both patients died within the observed 90 days (reward −100). Of note, in both cases, clinicians chose to apply an almost static ventilation regime over the entire 72 h observation period (Fig. 5a, b). In contrast, the VentAI algorithm dynamically explored a wide range of ventilator settings, which resulted in a reward of +96 and +98, respectively. Most notably, in both cases VentAI adjusted ventilator settings more frequently than clinicians (23 changes vs. 16 and 25 vs. 5, respectively). We further analyzed the importance of each feature included into the patient data fingerprint with respect to its impact in changing the chosen mechanical ventilation settings (Fig. 6a–c). We applied an out-of-bag analysis using random forest^10,17^. In fact, 19, 14, and 18 individual features constituted to 80% of the overall feature importance for choosing the optimized Vt_set_, PEEP, and FiO_2_ settings (Fig. 6a–c). This illustrates the wide range of impactful clinical parameters that are taken into consideration by VentAI. Of note, the weight of importance differed between the three dimensions of the action space (Vt_set_, PEEP, and FiO_2_).

Finally, we tested VentAI on an independent secondary dataset, comprising patient data for 200,859 patient distinct ICU stays with a total of 25,086 mechanical ventilation events (eICU dataset). In fact, the best dynamically chosen mechanical ventilation regime by the VentAI algorithm resulted in an estimated performance return of 84.1. In line with the findings from testing on MIMIC-III, the VentAI policy exceeded the relative action changes per mechanically ventilated patient indicating a similar dynamic algorithmic behavior (Supplementary Figs. 6–12).

## Discussion

In this study, we built VentAI based on 11,943 events of mechanical ventilation in order to dynamically support the attending physician in choosing an optimized mechanical ventilation policy for the individual patient state with the highest probability of 90-day or in-hospital survival. The algorithm provided reproducible high performance (on two independent datasets) in choosing the optimal ventilation policy. Most notably, the number of recommended action changes proposed by VentAI per mechanically ventilated patient consistently exceeded the number of action changes chosen by the clinicians. This indicates that VentAI might be of benefit in dynamically supporting the clinician’s decision making on individualized mechanical ventilation settings of the critically ill patient in order to achieve a personalized medicine within the ICU setting.

To date, the evidence for choosing an optimal mechanical ventilation regime is almost entirely determined by clinical studies. Other areas of medicine including genetics, cardiology, and radiology have a long (and strong) history of mathematical and engineering research that has been fundamental in driving significant advances in clinical care^9^. The lung of a patient suffering from acute respiratory failure has a very heterogeneous physiology^18,19^, with mixed healthy and diseased alveoli, displaying significant inter- and intra-patient variability. Thus, a mechanical ventilation protocol, which is highly effective in one patient may lead to VILI in another patient^20^. Consistent with other medical conditions, the real-world compliance to evidence-based recommendations for choosing the best mechanical ventilation regime, however, is often suboptimal. Clinicians tend to adjust the mechanical ventilation settings only infrequently and moderately during the clinical course of the patient^21^. In this retrospective analysis, we found a significantly increased estimated performance return of 83.3 (primary dataset) compared to physicians standard clinical care in the validation and testing dataset (51.1). In fact, the best dynamically chosen mechanical ventilation regime on the eICU dataset resulted in an estimated performance return of 84.1. Most notably, we observed, that the number of VentAI recommended action changes per mechanically ventilated patient constantly exceeded the number of action changes chosen by the clinicians over the whole observation period (Fig. 4a-c). These findings go in line with an animal study showing that the degree of variability of tidal volumes and respiratory frequency affects lung functional variables and hence, potentially improve patient outcomes^22^. It is important to acknowledge that a large part of the clinicians’ daily routine is covered by evaluating up to 1000 data points per patient per hour, also in order to choose the correct ventilation scheme. An algorithm evaluating those factors in a structured and reasonable manner, could potentially significantly cut down this time, hence free time for actual patient care (and ventilator adjustment) and reduce the burden on the treating medical personnel. This clearly indicates, that VentAI iteratively re-evaluates the optimal mechanical ventilation strategy throughout the course of the treatment while exploring a larger space of actions (Vtset, PEEP, FiO_2_) to find an optimized mechanical ventilation regime for the individual patient. It is important to underline that the used data from the MIMIC-III database includes data from 2001–2012. As the learnings from the mentioned trials are now broadly implemented into clinical practice, the physicians’ performance is likely to be closer to the VentAI algorithm with a newer database.

The consistently good performance of the VentAI algorithm (Fig. 2) can be explained by several attributes of a computerized ML approach: As the algorithm recognizes the full scope of the complex patients data fingerprint (including 44 features; Supplementary Table 1), it is able to categorize a patient’s individual development (state transition) faster and with finer granularity, compared to human physicians. Indeed, we found that 19, 14, and 18 features constituted 80% of the overall feature importance for choosing the optimized action (Fig. 6a-c). This clearly illustrates the wide range of impactful clinical parameters that are taken into consideration by VentAI, representing a holistic view of the patient’s status. The algorithm is able to compare the outcomes of a very detailed patient characteristic to a database of 11,943 mechanical ventilation events, predicting patient’s outcome precisely and consistently. As the attending physician is only able to compare the current patient’s status with a limited set of experienced scenarios (low amount of training data), the VentAI learning curve can be compared to the long-term experience of an extraordinarily experienced intensivist (high amount of training data). Given the availability of high computational power, the decision can be re-evaluated frequently, resulting in a highly dynamic system, repeatedly adapting the ventilation settings to the patients individual course and the optimal outcome^23^. While there is some general agreement on which mechanical ventilation settings and clinical parameters are preferred, there are several conflicting trial results^17,24–29^. Although focusing on patients with ARDS, the American Thoracic Society (ATS), the European Society of Intensive Care Medicine (ESICM), and the Society of Critical Care Medicine (SCCM) have recently endorsed clinical practice guidelines on mechanical ventilation in adult patients with ARDS. They suggest that an initial Vt should be set at 6 mL/kg predicted ideal body weight, while higher Vt should be avoided. Also in patients without ARDS, guidelines recommend the use of Vt of less than 8 mL/kg. The action space bins and their distribution are explained in detail in the Supplementary tables 2a and have been chosen with respect to the well accepted ventilation titration protocol published by the ARDS network as well as the S3 guideline on non-invasive ventilation^29^.

Our results strongly support this strategy but most importantly allow the treatment to be individualized for each patient in a constantly re-evaluating manner (Fig. 5). In fact, the VentAI policy chose more frequent ventilation regimes with lower Vt (5–7.5 mL/kg), but significantly less frequently regimes with higher Vt (10–12.5 mL/kg), compared to the clinician’s policy. Of note, high Vt settings of >15 ml/Kg were completely avoided by VentAI (Fig. 3, Tables 2 and 3). Taking two meta-analyses on different PEEP-levels into account^27,29^, there is clear evidence for the use of higher PEEP levels in patients with moderate or severe ARDS. However, adverse effects of PEEP, like cardiocirculatory instability and overexpansion of regional parts of the lung, make it still difficult to find individual PEEP settings. In line, VentAI chose significantly more often ventilation regimes with higher PEEP (5–7 cmH_2_O and 7–9 cmH_2_O) by avoiding very low PEEP settings (less than 5 cmH_2_O), compared to the clinicians. Most notably, however, the VentAI algorithm explored the PEEP-action space dynamically and extensively (Fig. 3, Tables 2 and 3, Supplementary Tables 2 and 3). As RL is an ML-approach to optimize sequences of decisions for long-term outcomes (e.g., 90-day survival), the choice of this toolset is ideal for decision making in longer observed timeframes, such as the treatment of critically ill patient^12^. However, RL-based approaches are not without limitations, and if used improperly, these approaches can replicate/suggest non-evidence based practices rather than improve the therapeutic strategy (and outcome) of the patients^12^.

Furthermore, importance sampling and off policy evaluation for reinforcement learning remain a challenge, especially in healthcare. Of note, there are alternatives to importance sampling such as Fitted Q Evaluation (FQE)^30^. One trade-off in off-policy learning is the fact that importance sampling is driven by mimicking the clinician policy. This can have negative implications in case of a suboptimal policy. On the other hand, not using importance sampling may eventually result in harmful recommendations^31–33^. In fact, in the context of healthcare, RL has recently been applied to different use cases, such as optimizing antiretroviral therapy in HIV^34^, modeling therapeutic strategy for epilepsy^35^, predicting time-to-extubation readiness^36^ and suggesting the optimal dose of fluids and vasopressors in sepsis therapy^10^. It is crucial to recognize that all these studies, including our work, are retrospective studies. Thus, some of the laboratory and clinical values retrospectively available to the algorithm, might not be immediately available in a prospective setting.

Furthermore, to estimate the value of a new action based on historical data, it is vital to take into account any information that was used by clinicians in their decision making in order to avoid estimates that are confounded by spurious correlation/relationships. Moreover, as MIMIC-III and eICU databases are exclusively derived from United States hospitals, these findings are not necessarily applicable to other countries. Local hospital policies and regional patient demographics are likely to have influenced the doctors’ performance in the observed patient datasets. Thus, additional verification of the algorithms’ performance on different multinational databases, including a more diverse dataset, is needed. Finally, assessment of the algorithm’s impact is necessary in a prospective setting designed to compare the clinical outcomes of the “treatment” group to a control group. Moreover, this study focuses on the “acute phase” of respiratory failure in the intensive care unit and is, thus, restricted to the first 72 h of the first mechanical ventilation event. Further work is needed to investigate the AI-policies in different phases of mechanical ventilation. The applied computational model could potentially be enhanced by conducting a manual analysis of the state’s specific characteristics from a medical perspective and projecting the outcome of this analysis on the reward function. Furthermore, a specific reward function (i.e., risk of VILI, etc.) might strengthen the directly related causation between ventilator settings and ventilation related outcome. By choosing to include a feature space of 44 included variables, we assured a broad applicability of the algorithm in the most common cases in the ICU. In special clinical situations, however, a deviation from these recommendations might be necessary (e.g., in cases of external oxygenation). It is important to underline, that in some cases, e.g., severe restrictive lung disease, it is impossible to reach a certain tidal volume. Although our algorithm focuses only on cases with volume-controlled ventilation, that are mostly sedated, there might be certain situations in which the algorithm’s suggested ventilation parameters cannot be implemented in clinical practice. Moreover, for the application to other ventilation modes (i.e., pressure-controlled modes), additional ventilation related variables, such as peak inspiratory pressure, have to be included. We plan to expand the algorithm in the future to other ventilation modes, as more reliable data sources become available. As the ability to reach a certain set level of tidal volume is also influenced by the current consciousness level, we decided to implement the Glasgow Coma Scale as a combined indicator of both pharmacological and pathophysiological (i.e. neurological disorders) reasons for an altered mental state. One advantage of VentAI is the ability to continuously observe a large feature space, which can draw new and unexpected clinical associations. This is a particularly important finding from the clinical perspective. An algorithm like VentAI continuously observes a multitude of clinical factors, weighing them individually for the patient case, trajectory and, most important, in a different pattern for each ventilation setting (PEEP, FiO_2_, and Vt). This means that even less acknowledged features, such as metabolic parameters or fluid status have to be taken into account, when choosing an optimal ventilation regime for a patient. For example, prothrombin time was found to be the second most influential feature highlighting the known association between coagulation abnormalities and acute lung injury and sepsis^37^. In summary, these findings clearly highlight the advantage of the usage of a computational algorithm like VentAI in the clinical routine, as the numbers of features that have to be taken into consideration clearly exceed the surveillance capacity of the treating physician or nurse.

As the aim of this work was to build an algorithm that is applicable in a wide range of clinical scenarios in hospital settings with variable technical abilities, we decided to include only features with a broad availability. Indeed, the algorithm could potentially be enhanced by providing additional confounders, which would lead to a more accurate presentation of the state space (e.g., pulmonary pressures, cardiac indices, image data, etc.). An observational study for this purpose must be based on a causal model validated by existing domain knowledge of medical experts. Further, it must also include well-known short-term indicators of deterioration in patient health, alongside long-term outcomes. Suitable alternatives to evaluate the performance of methods for estimating individual treatment effects in the mechanical ventilation setting would be to conduct a semi-synthetic simulation study^38,39^. Unfortunately, with the currently available dataset (MIMIC-III), we are unable to further stratify the cohort based on the Berlin criteria, as there is a lack of associated X-ray imaging for the observed cases as well as the information on potential cardiogenic cause for respiratory failure. However, as the X-ray data will become available in the upcoming release of MIMIC-IV database, we are already preparing the data preprocessing pipelines in order to further examine this mentioned aspect. However, the proposed computational model fits well with the problem statement as it is not possible to pick a no action/zero policy. In other words, clinicians and AI policies included an active setting for each decision time instant. This increases the validity of the performance comparison between clinicians and VentAI. Of note, the reduction in mortality on the test dataset is clear evidence that the algorithm is converging towards optimality. However, it is inaccurate to estimate the exact risk of 90-day mortality based on the VentAI performance return (Estimate of 90-day/in hospital mortality from return is presented in Supplementary Table 4). This is because VentAI is developed to optimize the probability of survival at 90 days, therefore, the mortality risk estimate, when VentAI is applied, might differ from the actually observed mortality rate. Addressing the high effect size in potential mortality reduction, we want to underline that from our perspective, this is not only the result of the correct ventilator settings alone but instead the result of an adapted, dynamic ventilation management, taking into account the whole status of the patient and the disease progression. Further, it is important to acknowledge that we apply a modern ventilation regime onto older datasets. Applying VentAI on a recent dataset would potentially show a smaller effect as modern guideline-adherent regimes are more widely adopted into practice. In conclusion, this study demonstrates the potential (on two independent datasets) of the application of VentAI, in the critical care domain, in particular in solving the complex and dynamical challenge of choosing the optimal mechanical ventilation regime. Rising computational power enables physicians to base medical decisions on patient-individual data patterns instead of simplified scoring systems. This might be particularly true for complex decision patterns, such as mechanical ventilation, because numerous clinical observations and data points must be considered when deciding on an optimal ventilation strategy. Special care must be taken when implementing decision-making tools based on RL algorithms into clinical routine. Patient safety can only be guaranteed with extensive clinical testing, taking aspects like algorithm bias, missing/false data, emergency situations and clinical particularities, such as rare diseases into account. Continuous monitoring of algorithmic performance must be implemented in order to maintain quality assurance. Until the long-term benefits and safety have been proven, the final decision on a complex task like mechanical ventilation will be in the physician’s hand and an algorithm like VentAI will stay a suggestive tool, thus highlighting the synergy between human and machine intelligence. Summarizing, computational algorithms, like the presented VentAI algorithm, will help to evaluate data fingerprints on a patient-individual basis and will likely be useful tools for decision making at the patient bed in intensive care medicine.

## Methods

### Study design

We built, validated and tested the performance of the VentAI algorithm on the MIMIC-III database, an open-access, anonymized database for ICU patients. The database contains data associated with 61,532 distinct ICU stays of adult patients admitted to the ICU of Beth Israel Deaconess Medical Center (Boston, MA, USA) between 2001 and 2012. We (repeatedly) randomly split the MIMIC-III database in three groups of 60% (training data), 20% (validation data), and 20% (testing data). Unlike the training set, the validation and testing sets are not used in establishing the model. Meanwhile, the testing set was used to quantify the performance of the policy with data never used in training or validation. Finally, we tested our findings on an independent, secondary dataset, eICU. This dataset contains data associated with 200,859 patient unit encounters for 139,367 unique adult patients admitted to 335 different ICUs in 208 teaching and nonteaching hospitals in the United States of America between 2014 and 2015. The overall methodological approach of this study is shown in Fig. 1.

### Patient cohort and data collection

61,532 and 200,859 ICU stays of adult patients are reported in the MIMIC-III and eICU datasets, respectively. An ICU stay has been created every time a patient is admitted to any ICU. This resulted in a specific unique ICU stay ID number, which refers to one single ICU stay. A single patient may have multiple ICU stays during the hospital stay, and all ICU stays are included in this study. The inclusion criteria for mechanically ventilated patients were the following: Age >18 years at the time of admission; treatment was not withdrawn within the assessed time frame; 90-day or in-hospital mortality was documented, mechanical ventilation for at least 24 h, and documented set tidal volume (Vt_set_). By focusing on a documented Vt_set_, we ensured the presence of a human-set target tidal volume, thus indicating a volume-controlled ventilation. This resulted in a total of 11,943 (MIMIC-III) and 25,086 (eICU) mechanical ventilation events, respectively. Data were collected for a period of 4 h before and 72 h after the onset of mechanical ventilation in 4-hour time steps. Patient demographics and clinical characteristics are shown in Table 1. This time window has been chosen based on the mean length of stay 6 (MIMIC: 3.1days (IQR 1.6–6.1); eICU: 3.0 (1.71–5.9)) in order to cover the majority of cases.

During preprocessing of the data, a mechanical ventilation event has been defined by applying the following criteria: The presence of a documented Vt_set_ starts a new ventilation event. The presence of a value of either Vt_set_, PEEP, or FiO_2_ during two sample periods (8 h) continued the event. The documentation of an extubation or the initiation of non-invasive ventilation and/or supplemental oxygen supply ends the current event. If multiple ventilation events were present during one single ICU stay, only the first event was included in the analysis. For training, validation, and testing, we collected a patient data fingerprint of 44 features for each patient included in the study (e.g. lab values, inputs/outputs, demographics) from both the MIMIC-III database and eICU database, extracted as multidimensional discrete time series in 4-hour time steps, averaged or summed as appropriate. As previously described, the features were selected according to their representativeness of the patient status and on clinical evidence towards the problem. Outliers were sorted out with univariate statistical approaches (Tukey’s range test) and frequency analysis (90% confidence interval). The observed primary outcome was the patients in-hospital or 90-day mortality.

### Data extraction

The extraction process has been performed by customized scripts (queries) of Standardized Query Language (SQL) for MIMIC and eICU on the object-relational database system PostgreSQL. The approval of data collection, processing and release for the eICU database has been granted by the eICU research committee and exempt from Institutional Review Board approval.

### Preprocessing steps

In time-varying datasets with high volume, one common practice for handling missing data is applying time-windowed sample-and-hold. In this method, a data point is simply repeated (held) to cover the available data point until either a new data point is available or the hold limit is reached. This limit protects the data from corruption by overholding a certain point. To choose the appropriate window size, we conducted a frequency analysis of the dataset and calculated an estimation of how frequently a new data point is produced. Thus, if the holding process goes further than this estimated limit, the data is corrupted with high probability^40^. Furthermore, k-nearest neighbor imputation^23^ with mean imputation and singular value decomposition (SVD), was adopted to handle the remaining missing data. If the preprocessing sample-and-hold resulted in over 50% missing data, the mechanical ventilation event was discarded (total incidence < 1% of the overall cohort). Notably, we tested the correlation between the data and the probability distribution of missing values for each of the 44 features. The feature Glasgow Coma Scale was associated with the highest p-value of 0.08. Thus, we were able to distinguish missing at random (MAR) from missing completely at random (MCAR) and not missed at random (NMAR) before proceeding further preprocessing steps^41^.

### Computational model

We used a Markov decision process (MDP), a discrete time stochastic control process, suitable for modeling decision problems, where outcomes are only partially under the decision makers control^42^. We projected our problem as MDP defined by the 4-tuple <S, A, T, R, γ > in the following sections.

### Model attributes

Assigned every 4 h, S is defined as a finite number of states, summarizing a patients clinical state (in total 650 different states) by clustering the patient’s data fingerprint (44 clinical features). In the clustering procedure, the state space was defined by clustering all patient time series from the MIMIC-III dataset. This was achieved using k-means clustering. Furthermore, we require a high value of k to ensure a highly granular model, while avoiding the usage of a too large state space. Thus, we adopted Bayesian and Akaike information criteria to determine the optimal number of clusters. This kept the state space away from having sparsely populated states. T is the transition matrix, describing the probability that an action A will lead in the next time step to state s^0^. γ is the discount factor, determining the weight of future rewards, regarding the current action. A high discount factor has the effect of resulting in a higher value of rewards received earlier than those received later in the decision process. Of note, a distribution of average return per patient in survivors and non-survivors is shown in Supplementary Fig. 1.

### Action space

The goals of a mechanical ventilation regime are the reduction of VILI while maintaining adequate oxygenation and decarboxylation. Consequently, we focused on a total of three parameters to be included in the action space, influencing these overall goals: Ideal body weight-adjusted (target) Vt_set_, PEEP, and FiO_2_. Ideal body weight-adjusted Vt was calculated relative to a predicted body weight for males as 50 + (0.91 × [height in centimeters − 152.4]) and for females as 45.5 + (0.91 × [height in centimeters − 152.4]). As a result, A is the finite number of possible actions at any given state based on a combination of the three aforementioned parameters: Vt_set_, PEEP, and FiO_2_. Based on frequency analysis, we divided the action space into three dimensions of seven treatment levels (bins), each representing a specific range of ventilator settings. This results in a multi-dimensional action space of 343 discrete actions. It is worth mentioning that there was no option of a zero policy and the algorithm always had to decide towards one ventilation policy. Of note, we analysed the effect of adding respiratory rate in the action space. Results related to the analysis of this added dimension are shown in the Supplementary.

### Reward system and patient trajectories

As there is strong evidence showing a direct link between VILI and mortality risk in critically ill patients, we decided on 90-day mortality as the primary reward in this study^3^. R is the given reward signal representing feedback received after the transition to a defined state (Supplementary Discussion). We modeled sequences of actions and states, so-called patient trajectories, using a reward/penalty system based on the patients 90-day mortality or the in-hospital mortality. Positive reward points of +100 were given to the trained model, if the patient survived, a penalty of −100 points assigned, if the patient died. As a result, a three-dimensional reward matrix R(s, s^0^, a) with current state s, next state s^0^ and action a, is computed by assigning the +100 or −100 values on the s^0^ dimension corresponding to a terminal state. Afterwards, this three-dimensional matrix is multiplied with the transition matrix T(s, s^0^, a) and summed over the dimension s^0^ to obtain R(s, a).

As the implementation of new policies (therapeutic strategies) in real-world patients may expose them to a not well-defined risk, we used off policy evaluation to assess the performance of a policy in a model-free manner^43^. We used Weighted Importance Sampling (WIS) to directly compare the policy performance of the VentAI algorithm to the performance of the attending physician. Of note, WIS is typically adopted in off-policy evaluation (OPE) problems such as ours. As long as the MDP is correctly specified, sequential exchangeability holds, and the observation policy is consistently estimated, it provides an accurate estimate of the performance of a trained policy without the need to execute it. In this regard, Important Sampling (IS) provides a way to decrease the differences between the learned policy (VentAI) and the observed policy (clinicians). This helps in decreasing the chance of suggesting a risky policy that may harm the patients. Additionally, we included a multiplicative control variate to reduce variance of the WIS estimate^44^. So-called off-policy evaluation, the evaluation of a certain policy given the behaviour data following a different policy, is used to evaluate the models performance. Of note, we conducted a correlation analysis of states versus time for each ICU stay within the observed 72 hours time period to observe separation between different disease states with respect to time (Supplementary Figs. 3 and 4).

### Learning scheme

In this work, we adopt Q-learning. This reinforcement learning algorithm fits well with our problem as it is a model-free algorithm, thus it does not require to learn the model of the environment. In MDP, Q-learning seeks to maximize the expected overall reward by tuning the treatment policy (Supplementary Discussion).

We generated 500 different models from various random splits (80%) of the MIMIC-III dataset. In each model, k-means clustering is performed to instantiate a different state space. Based on the Euclidean distance to the nearest cluster centroid, state membership and corresponding action for test set data points is determined. We then evaluated the AI policies using WIS on the remaining 20% of the data. Furthermore, we adopted bootstrapping on the validation dataset (20% from the 80% random split of the MIMIC-III dataset) in order to estimate the actual distribution of the policy value. This bootstrapping procedure offers confidence intervals for the WIS, and is adopted in wide range of high-risk applications^43,45^.

For each model, we estimate the value for the random policy. The selected final model maximizes the 95% confidence lower bound of the AI policy among the 500 candidate models.

### Ethics approval

Approval of data collection, processing, and release for the MIMIC-III database has been granted by the Institutional Review Boards of Beth Israel Deaconess Medical Center (Boston, MA, USA)^13^ and the Massachusetts Institute of Technology (Cambridge, MA, USA). Approval of data collection, processing and release for the eICU database has been granted by the eICU research committee and exempt from Institutional Review Board approval^14^. All data was processed on the computational infrastructure of the Rheinisch Westfälische Technische Hochschule (RWTH) Aachen University and the University Hospital RWTH Aachen in accordance to European Union data protection laws.

### Reporting summary

Further information on experimental design is available in the Nature Research Reporting Summary linked to this paper.

## Supplementary information

Supplementary Information

Reporting Summary

## Data Availability

The data that support the findings of this study are available from the corresponding author upon reasonable request. Access to the MIMIC-III and eICU database may be requested via: https://mimic.physionet.org/ and https://eicu-crd.mit.edu.

## Code availability

The full code generated to produce this work is available via the dedicated VentAI-website https://ventai.org.
