# Supplementary material for: Development and validation of a reinforcement learning algorithm to dynamically optimize mechanical ventilation in critical care
Source: NPJ Digit Med. 2021 Feb 19;4:32. doi: 10.1038/s41746-021-00388-6 (PMC7895944; doi:10.1038/s41746-021-00388-6)
Supplement: Supplementary file 1 — Supplementary Information [file 41746_2021_388_MOESM1_ESM.pdf]

Supplementary Information

Supplementary tables:

1. Table 1: Patient data fingerprint / extracted feature set in MIMIC-III and eICU database 2
2. Tables 2a/b/c: Frequency distribution within the action space on MIMIC-III dataset
    a. Distribution of the chosen action by clinicians on MIMIC-III dataset 3
    b. Distribution of the chosen action by VentAI on MIMIC-III dataset 3
    c. Relative differences in decision between VentAI and clinicians on MIMIC-III dataset 3
3. Tables 3a/b/c: Frequency distribution within the action space on eICU dataset
    a. Distribution of the chosen action by clinicians on eICU dataset 4
    b. Distribution of the chosen action by VentAI on eICU dataset 4
4. Table 4: Relationship between return of action and 90-day mortality or in-hospital mortality 5
5. Table 5: Subcohort analyses on clinical and demographic properties 6
6. Table 6: Comparison of weights between out-of-bag feature for TV, PEEP and FiO2 7

Supplementary figures:

1. Figure 1: Distribution of average return per patient in survivors / non-survivors in MIMIC-III dataset 9
2. Figure 2: Distribution of death or release from ICU within the observed time window 72 hours in MIMIC-III dataset 10
3. Figure 3: Correlation of all 650 states with decay function in MIMIC-III dataset 11
4. Figure 4: Correlation of patient population across states over time in MIMIC-III dataset 12
5. Figure 5: Visualization of the action distribution in the 3-dimensional action space on eICU dataset 13
6. Figure 6: Number of action changes on eICU dataset 14
7. Figure 7: Out-of-Bag feature weight analysis of VentAI on eICU dataset 15
8. Figure 8: Flow-chart of studied cohort 16
9. Figure 9: Estimated performance return of random policy on MIMIC-III dataset 17
10. Figure 10: Visualization of the action distribution in the Respiratory rate dimension (MIMIC-III dataset) 18
11. Figure 11: Out-of-Bag feature weight analysis for Respiratory Rate in VentAI (MIMIC-III dataset) 19
12. Figure 12: Number of action changes for Respiratory rate of ventAi compared to Clinicians (MIMIC-III dataset) 20
13. Figure 13: Visualization of one representative patient case (MIMIC-III dataset) 21

Supplementary Discussion 22

Supplementary References 23

Supplementary Table 1: Patient data fingerprint in MIMIC-III and eICU database.

As previously described[1], for each included patient, we collected a "patient data fingerprint" of 44 features (e.g. lab values, inputs/outputs, demographics) from the MIMIC-III and eICU database[2,3], extracted as multidimensional discrete time series in 4 hour time steps, averaged or summed as appropriate.

Demographics	Age, Gender, Weight, Readmission to ICU-Elixhauser Premorbidity score
Vital signs	SOFA, SIRS, GCS, HR, RRsys, MAP, RRdys, shock index, Respiratory rate, SpO2, Temperature
Lab values	Potassium, sodium, chloride, Glucose, BUN, creatinine Magnesium, calcium, ionized calcium, carbon dioxide, total bilirubin, albumin Hemoglobin, White blood cells count, platelets count, PTT, PT, INR, pH, PaO2, PaCO2, base excess, bicarbonate, lactate, PaO2/FiO2 ratio
Medications and fluid balance	intravenous fluid intake over 4h, maximum dose of vasopressor over 4h, Urine output over 4h, cumulated fluid balance since admission (including preadmission data when available)
Outcome	Hospital mortality, 90-day mortality

Supplementary Table 1: Patient data fingerprint. ICU: intensive care unit; SOFA: Sequential Organ Failure Assessment Score, SIRS: Systemic Inflammatory Response Syndrome; GCS: Glasgow Coma Scale; HR: heart rate per minute; RRsys: systolic blood pressure; MAP: mean arterial pressure; RRdys: diastolic blood pressure; hock index: systolic blood pressure/heart rate, BUN: blood urea nitrogen; PTT: Partial Thromboplastin Time; PT: Prothrombin Time; INR: International Normalized Ratio.

Supplementary Table 2a: Distribution of the chosen action by clinicians on MIMIC-III dataset.

35,556 total decision time instances, bin ranges are distributed as follows from 1-7:

TV [mL/Kg]	0-2.5, 2.5-5, 5-7.5, 7.5-10, 10-12.5, 12.5-15, >15 [mL/Kg]
PEEP [cmH2O]	0-5, 5-7, 7-9, 9-11, 11-13, 13-15, >15 [cmH2O]
FiO2 [%]	25-30, 30-35, 35-40, 40-45, 45-50, 45-50, 50-55, >55 [%]

	1	2	3	4	5	6	7
FiO2	269	751	3751	9880	16789	1854	2262
PEEP	16154	13819	2810	1992	436	294	51
TV	1	47	6161	22452	6078	686	131

Supplementary Table 2b: Distribution of the chosen action by VentAI on MIMIC-III dataset.

35,556 total decision time instances, Bins are distributed as described in Figure 2a.

	1	2	3	4	5	6	7
FiO2	316	382	2998	8811	17684	4456	909
PEEP	11755	17868	4318	1097	425	16	77
TV	219	2453	18665	11037	3072	686	131

Supplementary Table 2c: Relative differences in decision between VentAI and clinicians on MIMIC-III dataset.

A total of 35,556 total decision time instances, bins are distributed as described in S2a. Positive numbers mean that an action has been chosen more frequently by VentAI, negative numbers represent an action chosen less frequently. In comparison to table 2b of the full publication, incidents with less than 1% from the total possible time decision incidence are included.

	1	2	3	4	5	6	7
FiO2	47	-369	-753	-1069	895	2602	-1353
PEEP	-4399	4049	1508	-895	-11	-278	26
TV	218	2406	12504	-11415	-3006	-576	-131

Supplementary Table 3a: Distribution of the chosen action by clinicians on eICU dataset. 378,006 total decision time instances, bin ranges are distributed as follows from 1-7:

TV [mL/Kg]	0-2.5, 2.5-5, 5-7.5, 7.5-10, 10-12.5, 12.5-15, >15 [mL/Kg]
PEEP [cmH2O]	0-5, 5-7, 7-9, 9-11, 11-13, 13-15, >15 [cmH2O]
FiO2 [%]	25-30, 30-35, 35-40, 40-45, 45-50, 45-50, 50-55, >55 [%]

	1	2	3	4	5	6	7
TV	180	1163	177883	187151	10747	745	137
PEEP	209179	112694	32476	17643	6014	0	0
FiO2	58920	47564	144721	42563	46097	8034	30107

Supplementary Table 3b: Distribution of the chosen action by VentAI on eICU dataset. 378,006 total decision time instances, Bins are distributed as described in S3a.

	1	2	3	4	5	6	7
TV	2849	1280	109637	237197	27043	0	0
PEEP	135438	179970	46804	15439	355	0	0
FiO2	3159	4948	34509	115052	179123	33044	8171

Supplementary Table 4: Relationship between return of action and 90-day mortality or in-hospital mortality (MIMIC-III dataset). A low return is associated with a high probability of mortality and vice versa, further underlining the validity of the reward function.

Return of action	Mortality Risk
-100	1.0292
-90	0.08959
-80	0.7898
-70	0.7257
-60	0.6843
-50	0.6401
-40	0.5914
-30	0.5513
-20	0.507
-10	0.4715
0	0.4341
10	0.3982
20	0.3575
30	0.3094
40	0.2732
50	0.2331
60	0.1913
70	0.1535
80	0.1142
90	0.028
100	0.0075

Supplementary Table 5: Subcohort analyses on clinical and demographic properties.

Demographic and clinical data of two patient sub-cohorts on admission to the ICU. The total cohort (all) was divided into two sub-cohorts according to their ventilation strategies. Cohort 1 consists of patients where clinicians chose to significantly deviate from the common clinical practices, indicated by choosing a high VTset (> 10mL/kg), high/low FiO2 (>55% and <40%) and high/low PEEP (>9 cmH2O and <5 cmH2O). Cohort 2 consists of all other included patients extracted from the MIMIC III database. Data is presented in n (%), mean (SD) or median (IQR); LOS: Length of stay, SOFA: Sequential Organ Failure Assessment Score.

**Title**	**All**	**Cohort 1**	**Cohort 2**
Number of distinct ICU admissions	11.943	4.207	7.736
Age, years	66.9 (56.3-77.5)	67.6 (46.8-88.3)	66.9 (45.7-88.1)
Body weight, kilogram	85.7 (±18.1)	89.4 (±18.7)	84.8 (±18.4)
SIRS, points	1.1 (±0.9)	1.2 (±0.8)	1.1 (±0.9)
Elixhauser score, points	6.7 (±3.1)	6.6 (±3.7)	6.8 (±3.0)
In-hospital mortality, %	11.1	11.6	11.0
SOFA, points	5.6 (±2.9)	6.1 (±2.8)	5.5 (±2.9)

Supplementary Table 6: Comparison of weights between out-of-bag feature for Vt, PEEP and FiO2 (MIMIC III dataset): The relative increase or decrease of the weights of out-of-bag feature analysis of positive end expiratory pressure (PEEP) and fraction of inspired oxygen (FiO2) compared to the weights of ideal body weight-adjusted tidal volume (Vt)

Out-of-bag weight analysis factors of **Vt**	Relative change w.r.t.**PEEP**	Relative change w.r.t. **FiO2**
Age	Increase	Equal
MV	Decrease	Equal
MeanBP	Decrease	Decrease
Bilirubin	Decrease	Decrease
Fluid Balance	Decrease	Decrease
Potassium	Decrease	Decrease
Weight	Decrease	Decrease
Magnesium	Decrease	Decrease
PT	Decrease	Decrease
Cumulated input	Decrease	Decrease
WBC	Decrease	Decrease
RR	Decrease	Decrease
Shock Index	Decrease	Decrease
Sodium	Equal	Decrease
BE	Decrease	Decrease
Lactate	Decrease	Equal

PTT	Decrease	Decrease
INR	Increase	Decrease
PaO2/FiO2	Increase	Increase

Supplementary Figure 1: Distribution of average return per patient in survivors/non-survivors in training dataset in MIMIC-III dataset. The distribution of the average return within the survivors shows two clearly distinguishable groups within survivors / non-survivors with a near Gaussian distribution.

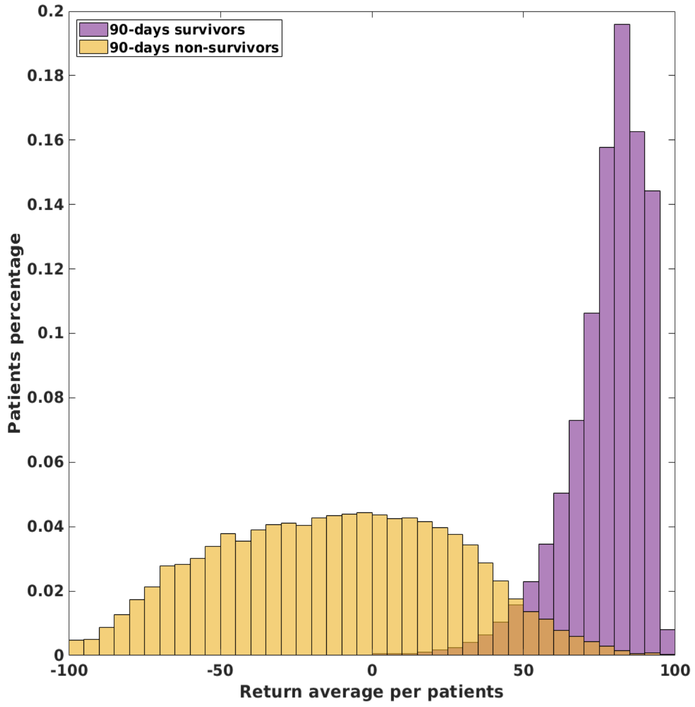

Supplementary Figure 2: Distribution of death or release from ICU within the observed time period (72 hours) in MIMIC-III dataset. An analysis of the leaves from ICU shows a periodicity of overall leaves while the ratio of mortal leaves stays constant.

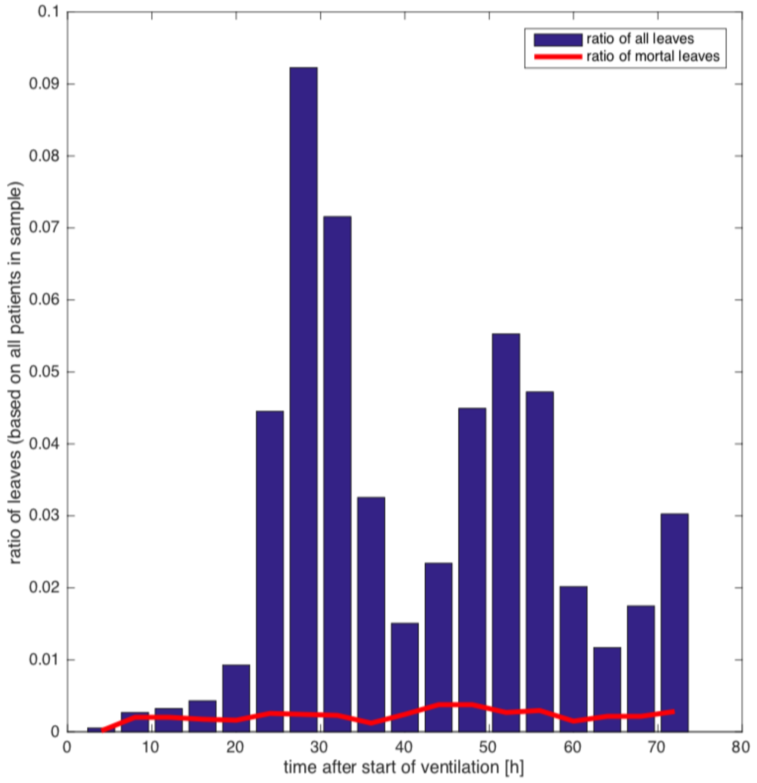

Supplementary Figure 3: Correlation of all 650 states with decay function in MIMIC-III dataset

One indication for fulfilling the Markovian criteria needed for the overall scheme is to observe the lifespan of each state and monitor its correlation with a decay function. Accordingly, we next conducted this analysis for the 650 states. The correlation shows a r2-value of 99.9%, thus providing evidence for the soundness of the model.

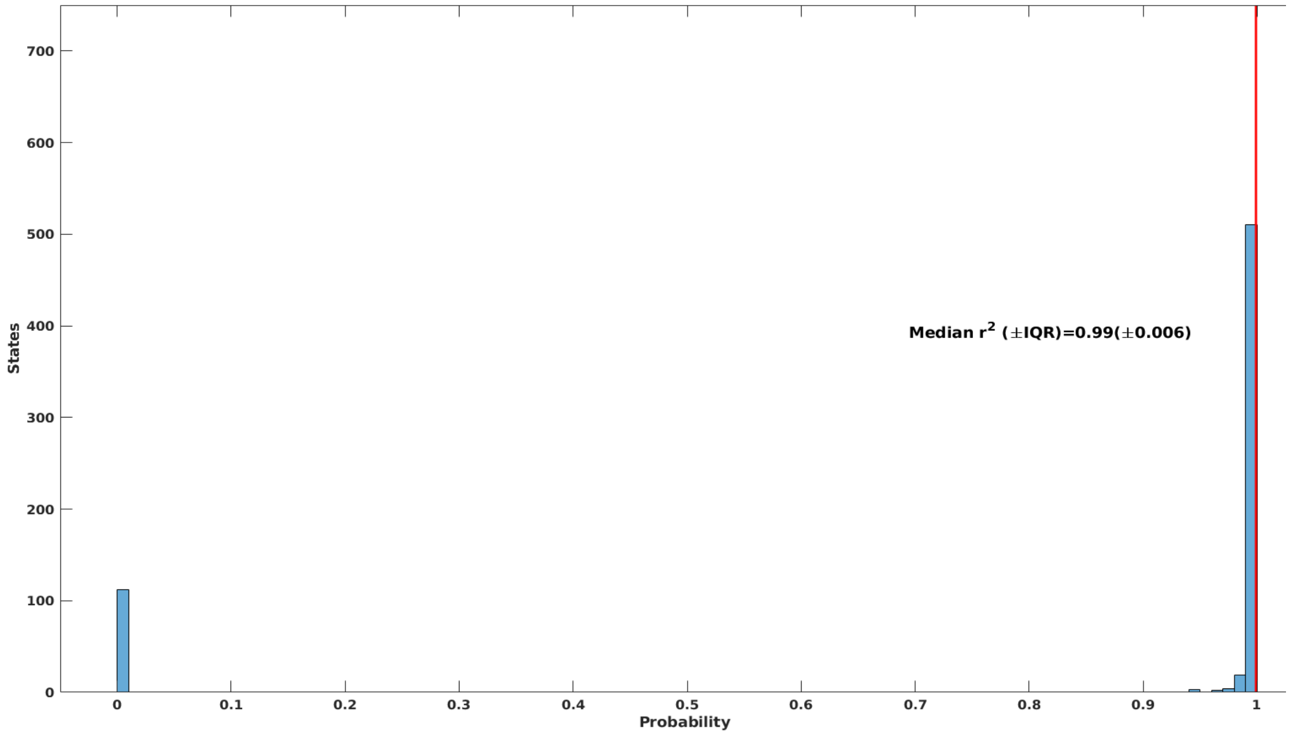

Supplementary Figure 4: Correlation of patient population across states over time in MIMIC-III dataset

We analyzed the correlation function C(t,t′)= corr(P(s,t), P(s,t′) of states versus time for each ICU stay within the observed 72 hours time period. As clearly indicated by the distribution of the correlation, we observed an existence of an overall steady state beyond 40 hours after start of ventilation. This indicates a clear phase separation (early: 0 - 40 hours, late 40 - 72 hours) in the population of disease states, thus giving a clear indication of fulfillment of the Markov criteria in the late phase.

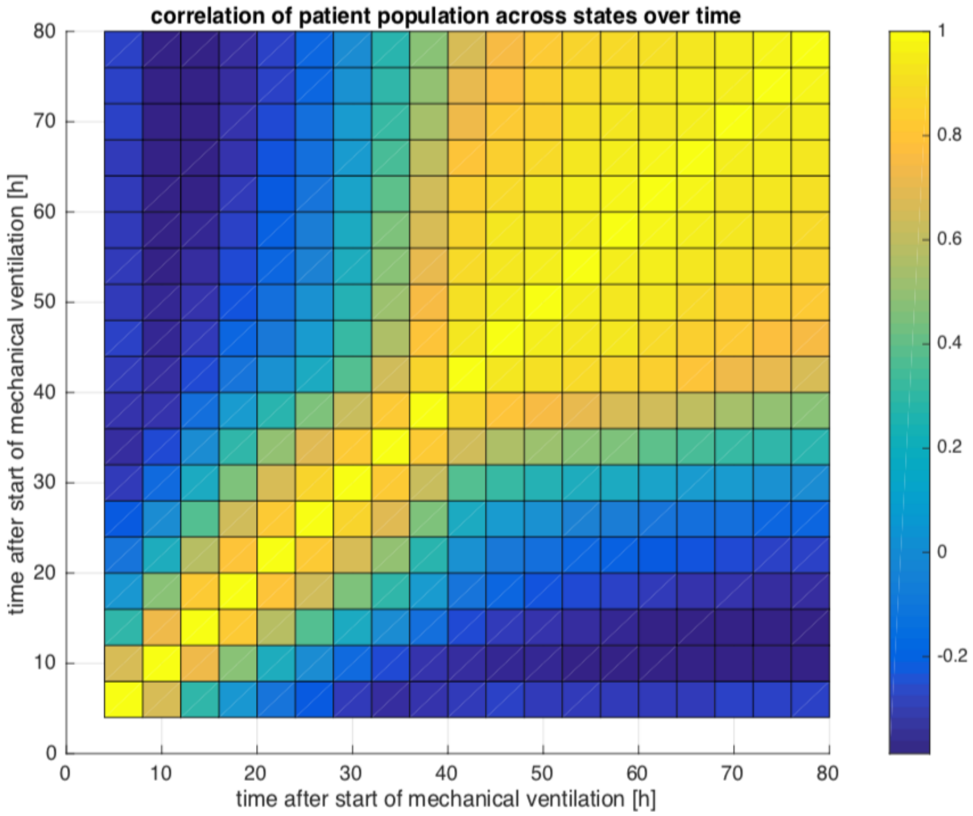

Supplementary Figure 5: Visualization of the action distribution in the 3-dimensional action space on eICU dataset.

The distribution of the chosen actions (ideal body weight-adjusted tidal volume (Vt), positive end expiratory pressure (PEEP), and fraction of inspired oxygen (FiO2)) by clinicians (left) and by the best 90% lower bound VentAI policy (right) on eICU dataset. The eICU dataset includes 378,006 decision time instances and the designed model facilitates 343 action bins in the action space.

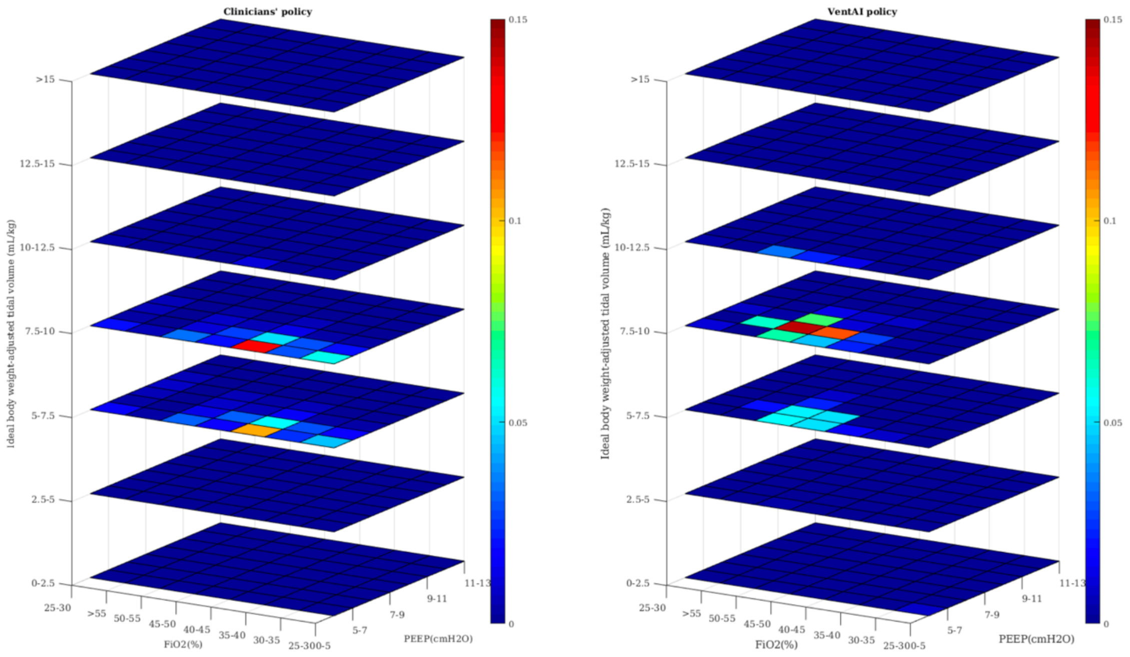

Supplementary Figure 6: Number of action changes on eICU dataset.

The relative number of action changes (ideal body weight-adjusted tidal volume (Vt), positive end expiratory pressure (PEEP), and fraction of inspired oxygen (FiO2)) is shown in relation to the number of mechanically ventilated patients at each 4 hour time step. Clinicians action changes are shown in blue while the VentAI action changes are shown in red.

A Ideal body weight-adjusted tidal volume (mL/kg)

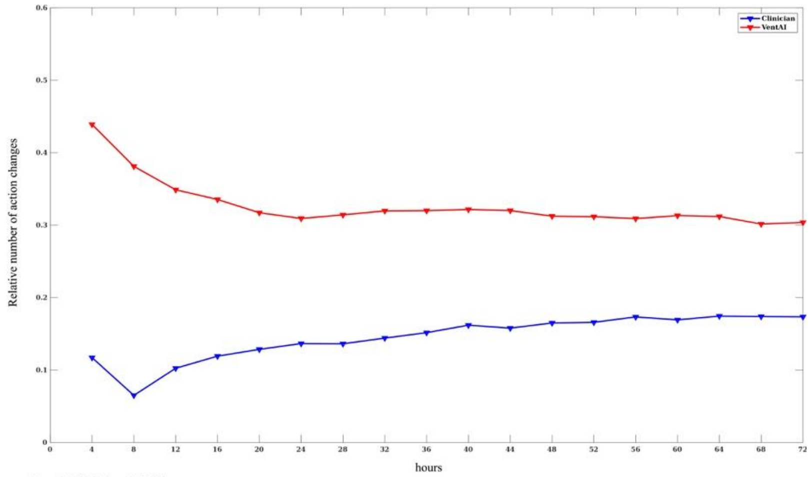

B PEEP (cmH2O)

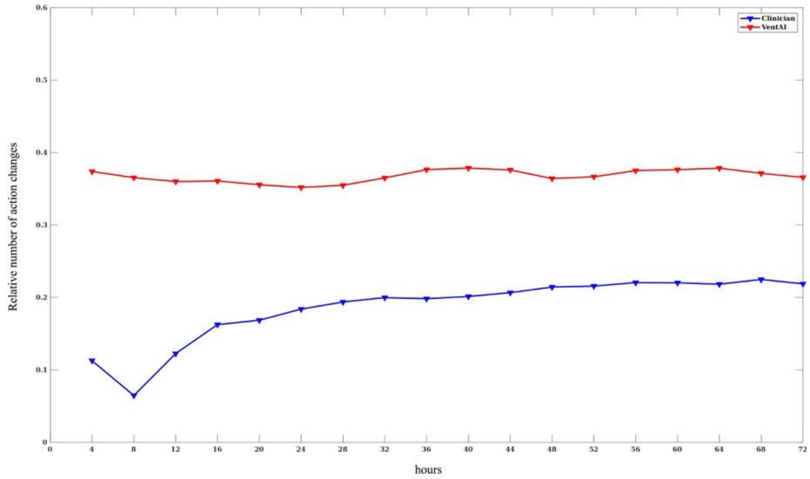

C FiO2 (%)

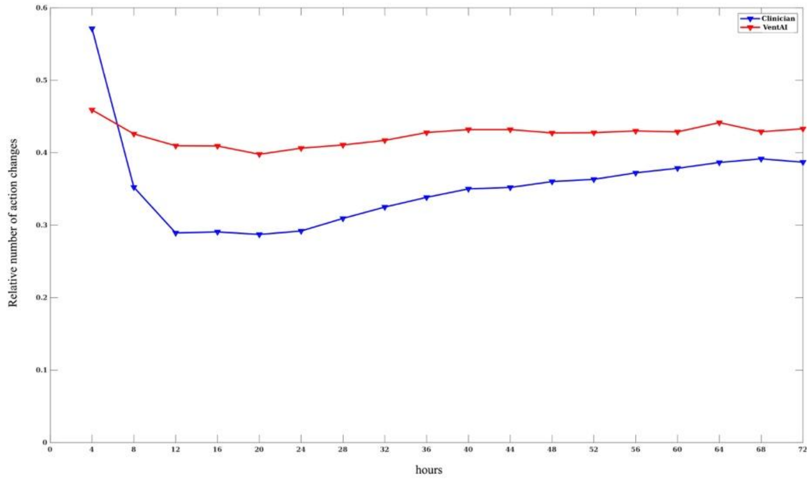

Supplementary Figure 7: Out-of-Bag feature weight analysis of VentAI on eICU dataset.

Relative weight of each feature using out-of-bag feature weight analysis, based on the relative loss of prediction, represented by an increase of the mean squared error. ICU: intensive care unit; SOFA: Sequential Organ Failure Assessment Score, SIRS: Systemic Inflammatory Response Syndrome; GCS: Glasgow Coma Scale; HR: heart rate per minute; RRsys: systolic blood pressure; MAP: mean arterial pressure; RRdys: diastolic blood pressure; shock index: systolic blood pressure/heart rate, BUN: blood urea nitrogen; PTT: Partial Thromboplastin Time; PT: Prothrombin Time; INR: International Normalized Ratio.

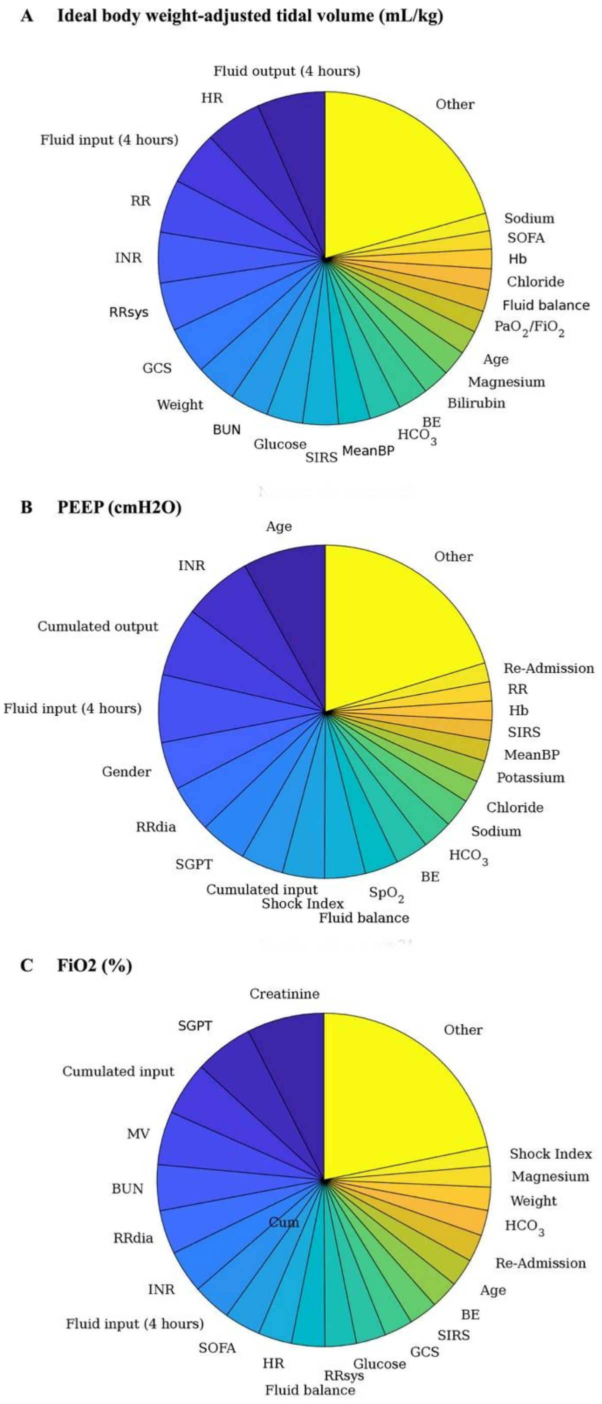

Supplementary Figure 8: Flowchart of studied cohort.

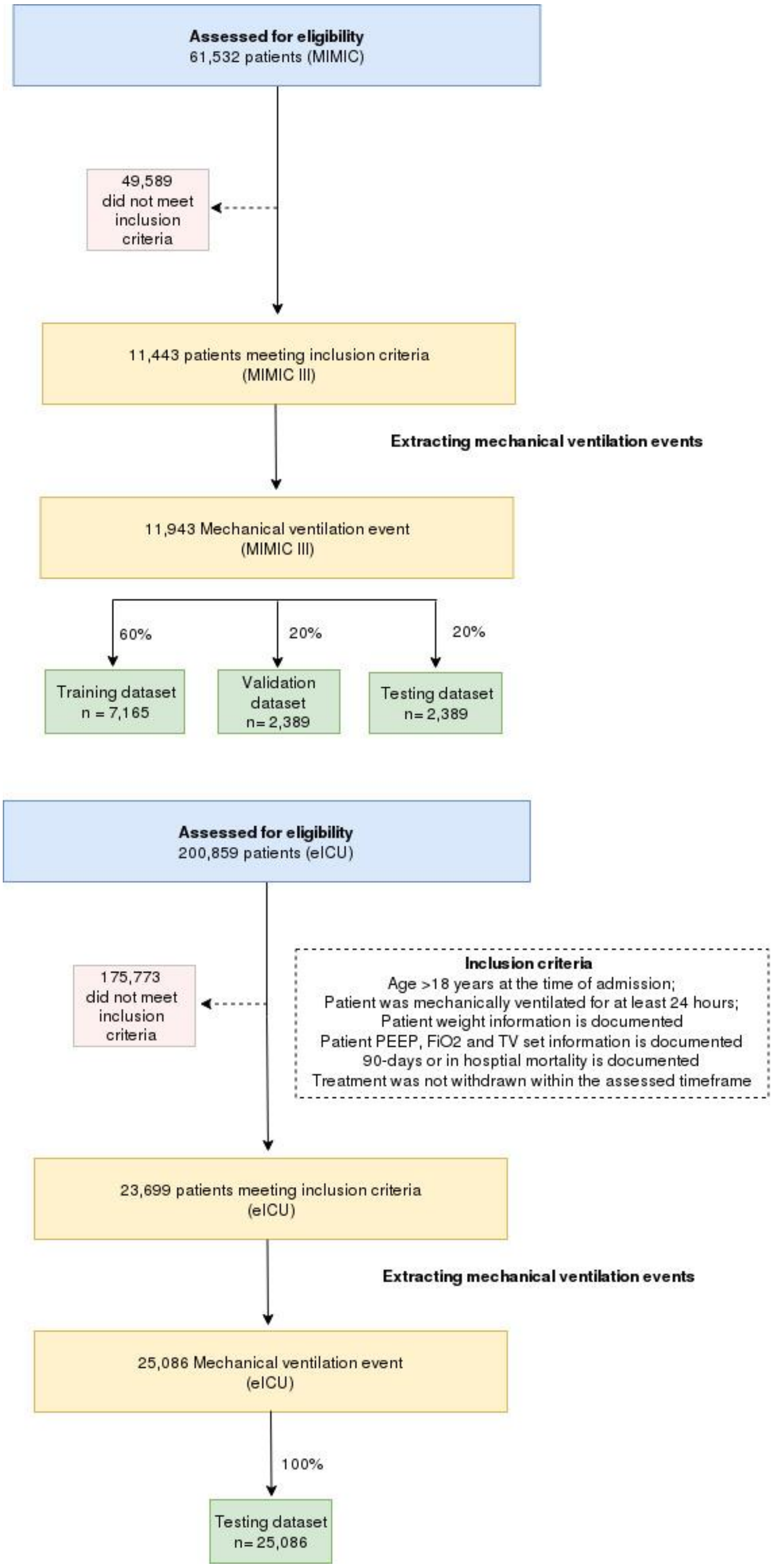

Supplementary Figure 9: Estimated performance return of random policy on MIMIC-III dataset.

The estimated performance return after the exposure of random policy to 500 models. The purple line represents the cumulative average of the estimated performance return of a random policy model on the MIMIC-III dataset. The grey lines represent the precise values.

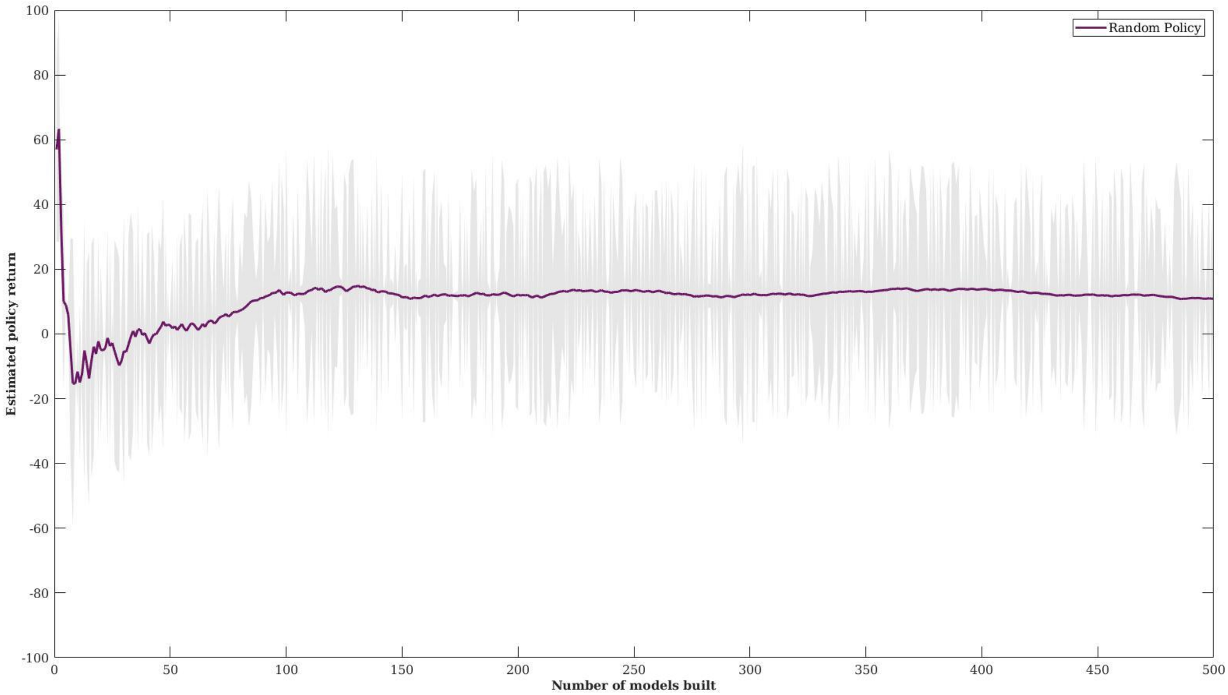

Supplementary Figure 10: Visualization of the action distribution in the Respiratory rate dimension (MIMIC-III dataset)

We elucidated the frequency distributions of the chosen optimal VentAI actions in the Respiratory rate dimension, compared to the clinicians, after conducting evaluations on 500 models. We performed a detailed frequency analysis on the RR action dimension. This analysis revealed that the VentAI algorithm chose similar ventilation regimes with respect to RR.

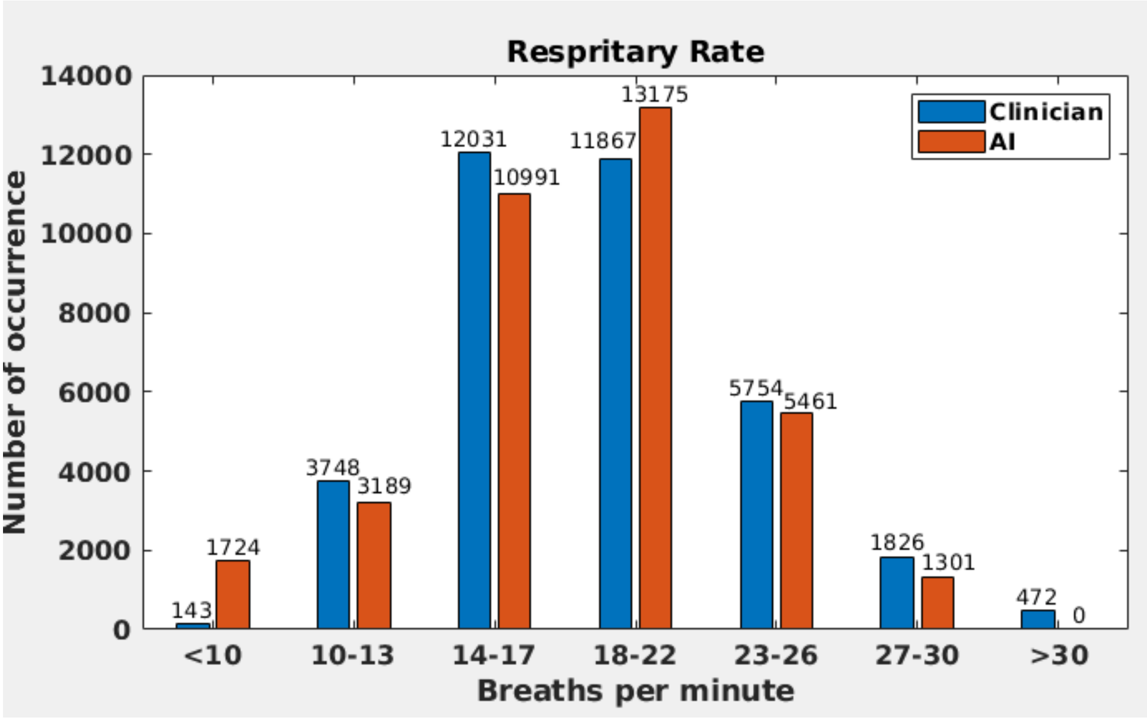

Supplementary Figure 11: Out-of-Bag feature weight analysis for Respiratory Rate in VentAI (MIMIC-III dataset)

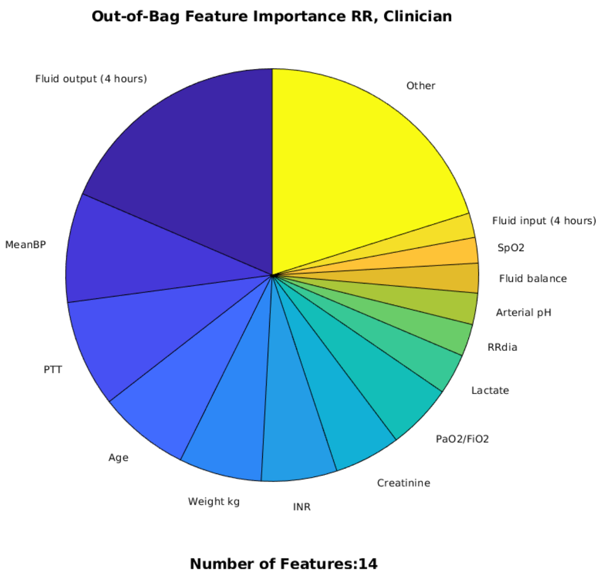

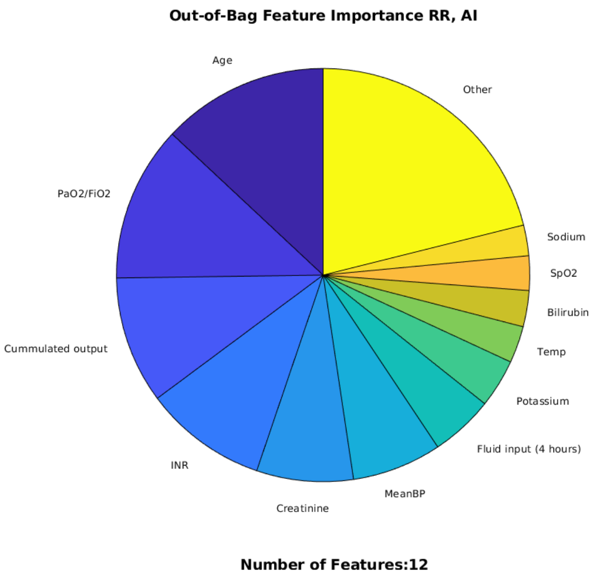

Supplementary Figure 12: Number of action changes for Respiratory rate of VentAI compared to Clinicians (MIMIC-III dataset): The relative number of action changes Respiratory rate is shown in relation to the number of mechanically ventilated patients at each 4 hour time step. Clinicians action changes are shown in blue while the VentAI action changes are shown in red

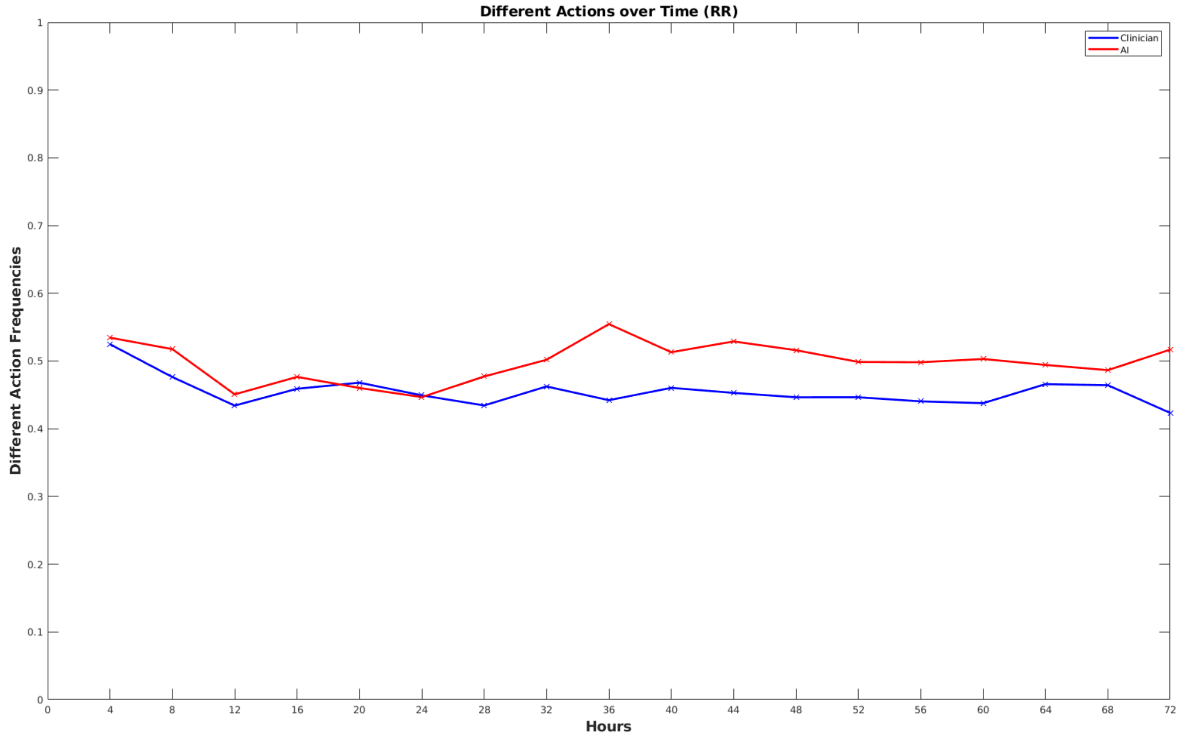

Supplementary Figure 13: Visualization of one representative patient case (MIMIC-III dataset)

Visualization of one representative case studies in 4-hour intervals. Clinicians' actions are shown in blue while the VentAI actions are shown in red.

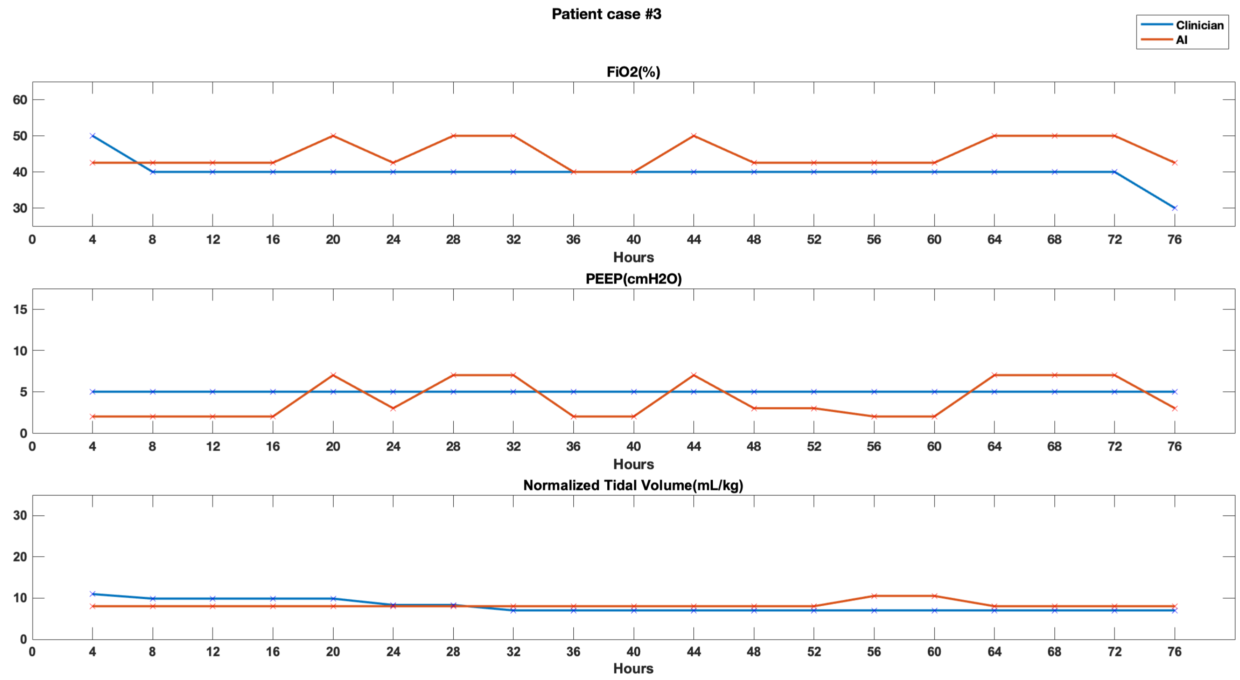

Supplementary Discussion:

A: Evaluation of policies

The three-dimensional reward matrix R(s, s′, a) with current state s, next state s′and action a, is computed by assigning the +100 or -100 values on the s′dimension corresponding to a terminal state. Afterwards, this three-dimensional matrix is multiplied with the transition matrix T(s, s′, a) and summed over the dimension s′ to obtain R(s, a). The transition matrix can be calculated as follows using the available date (1):

$$T(s, s', a) = \Pr\left(S_{t+1} = s' | S_t = s, A_t = a\right) = \frac{\Pr\left(S_{t+1} = s', S_t = s, A_t = a\right)}{\Pr\left(S_t = s, A_t = a\right)}$$

The recommended action to take for each given state is called a policy (here, a specific ventilator setting). The value of the policy is determined by the likelihood of survival under the given policy. Of note, for clinician's policy evaluation, we needed a model-free learning procedure to facilitate learning from sample trajectory. As a result, we adopted temporal difference (TD) learning of the Q function based on offline sampling on the available recorded actions of the clinicians. Thus, using TD reinforcement Q-learning algorithm, we started with an initial state and arbitrary approximation of the Q-function. We chose a fitted Q iteration-based method.[18] This estimation was updated using the reward from the next transition by Bellman recursion for Q-values, taking the relative weight of current and previous estimations into consideration.[19] The update of the Q-value is presented in the following equation (2):

$$\underbrace{Q^{\pi}(s,a)}_{\text{new}} \leftarrow \underbrace{Q^{\pi}(s,a)}_{\text{old}} + \overbrace{\alpha}^{\text{learning rate}} \left[\underbrace{\mathcal{R}(s,a)}_{\text{reward function}} + \underbrace{\gamma}_{\text{discount factor}} \max_{a} Q(s\prime, a) \right]$$

Where π is the policy and Q^{π} is the Q-value for policy π. Please note that we adopted a discount factor of value 0.99. On the other hand, for VentAi, the objective of the learning process is to find the optimal policy that maximizes the overall expected discounted reward. The value of a policy is calculated as follows (3):

$$\underbrace{\mathcal{V}^{\pi}(s)}_{\text{value of a policy}} \leftarrow \sum_{a} \pi(s,a) \sum_{s\prime} \underbrace{\mathcal{T}(s\prime, s, a)}_{\text{transition matrix}} \left[\underbrace{\mathcal{R}(s,a)}_{\text{reward function}} + \underbrace{\gamma}_{\text{discount factor}} \mathcal{V}^{\pi}(s\prime) \right]$$

B: Weighted Importance Sampling

The first step is to calculate the per-step importance ratio. At time t, importance ratio is (4):

$$\eta = \pi(a_t, s_t)/\pi_0(a_t, s_t)$$

Where π is VentAi policy and π_0 is Clinician's (observed policy). This can be then used to calculate the cumulative importance ratio for a horizon for time t as follows (5):

$$\eta_{1:t} = \Pi_{t=1}^{t} \eta_t$$

Consequently, the average importance ratio until time t can be calculated by (6):

$$W_t = \sum_{1}^{|D|} \eta_{1:t}^{(i)}/|D|$$

Where D is the dataset and |D| is the number of trajectories. Furthermore, the weighted importance sampling estimation per trajectory can be calculated as follows (7):

$$T_{wis} = \frac{\eta_{1:E}}{W_t} \sum_{t=1}^{E} \gamma^{t-1} r_t$$

Where E is the ending trajectory sample. Finally, the overall estimator for all trajectories in D is (8):

$$\Theta_{wis} = \frac{1}{|D|} \sum_{k=1}^{|D|} T_{wis}^{(i)}$$

Of note, we adopted bootstrapping on the estimator by randomly picking a patient from the test set 2000 times and use the 90% lower bound as the estimator value. Furthermore, we have adopted the same state clustering output across all evaluations instead of changing it based on the policy being evaluated. Using the same cluster in the estimation of π_0 stopped potential bias to select the clustering that is favoring the learned policy.

Supplementary References

1 Komorowski M, Celi LA, Badawi O, Gordon AC, Faisal AA. The Artificial Intelligence Clinician learns optimal treatment strategies for sepsis in intensive care. *Nat Med* 2018; **24**: 1716.

2 Johnson AEW, Pollard TJ, Shen L, *et al*. MIMIC-III, a freely accessible critical care database. *Sci Data* 2016; **3**: 160035.

3 Pollard TJ, Johnson AEW, Raffa JD, Celi LA, Mark RG, Badawi O. The eICU Collaborative Research Database, a freely available multi-center database for critical care research. *Sci Data* 2018; **5**: 180178.

4 Sutton, Richard S., and Andrew G. Barto. *Introduction to reinforcement learning*. Vol. 135. Cambridge: MIT press, 1998.
