# Supplementary material for: Development and validation of a reinforcement learning algorithm to dynamically optimize mechanical ventilation in critical care
Source: NPJ Digit Med. 2021 Feb 19;4:32. doi: 10.1038/s41746-021-00388-6 (PMC7895944; doi:10.1038/s41746-021-00388-6)
Supplement: Supplementary file 2 — Reporting Summary [file 41746_2021_388_MOESM2_ESM.pdf]

nature research

Corresponding author(s): Priv.-Doz- Dr. med. Lukas Martin, MHBA

Last updated by author(s): 2021.01.07

Reporting Summary

Nature Research wishes to improve the reproducibility of the work that we publish. This form provides structure for consistency and transparency in reporting. For further information on Nature Research policies, see our Editorial Policies and the Editorial Policy Checklist.

Statistics

For all statistical analyses, confirm that the following items are present in the figure legend, table legend, main text, or Methods section.

n/a	Confirmed	
☒	☐	The exact sample size (*n*) for each experimental group/condition, given as a discrete number and unit of measurement
☒	☐	A statement on whether measurements were taken from distinct samples or whether the same sample was measured repeatedly
☒	☐	The statistical test(s) used AND whether they are one- or two-sided<br>*Only common tests should be described solely by name; describe more complex techniques in the Methods section.*
☒	☐	A description of all covariates tested
☐	☒	A description of any assumptions or corrections, such as tests of normality and adjustment for multiple comparisons
☐	☒	A full description of the statistical parameters including central tendency (e.g. means) or other basic estimates (e.g. regression coefficient) AND variation (e.g. standard deviation) or associated estimates of uncertainty (e.g. confidence intervals)
☒	☐	For null hypothesis testing, the test statistic (e.g. *F*, *t*, *r*) with confidence intervals, effect sizes, degrees of freedom and *P* value noted<br>*Give P values as exact values whenever suitable.*
☒	☐	For Bayesian analysis, information on the choice of priors and Markov chain Monte Carlo settings
☐	☒	For hierarchical and complex designs, identification of the appropriate level for tests and full reporting of outcomes
☒	☐	Estimates of effect sizes (e.g. Cohen's *d*, Pearson's *r*), indicating how they were calculated

Our web collection on statistics for biologists contains articles on many of the points above.

Software and code

Policy information about availability of computer code

Data collection	The databases were queried in pgAdmin 4 v 1.2. Computations were implemented in Matlab R2018b (MathWorks Inc., Natick, MA).
Data analysis	Matlab R2018b (MathWorks Inc., Natick, MA).

For manuscripts utilizing custom algorithms or software that are central to the research but not yet described in published literature, software must be made available to editors and reviewers. We strongly encourage code deposition in a community repository (e.g. GitHub). See the Nature Research guidelines for submitting code & software for further information.

Data

Policy information about availability of data

All manuscripts must include a data availability statement. This statement should provide the following information, where applicable:

- Accession codes, unique identifiers, or web links for publicly available datasets
- A list of figures that have associated raw data
- A description of any restrictions on data availability

The extraction process has been performed by customized scripts (queries) of Standardized Query Language (SQL) for MIMIC and eICU on the object-relational database system PostgreSQL. The full SQL source code is provided via the dedicated VentAI website: https://ventai.org

Field-specific reporting

Please select the one below that is the best fit for your research. If you are not sure, read the appropriate sections before making your selection.

☒ Life sciences ☐ Behavioural & social sciences ☐ Ecological, evolutionary & environmental sciences

For a reference copy of the document with all sections, see nature.com/documents/nr-reporting-summary-flat.pdf

Life sciences study design

All studies must disclose on these points even when the disclosure is negative.

Sample size	We included all adult patients from two large intensive care databases, MIMIC-III and eICU-RI. We conducted a secondary analysis of patient data initially collected routinely for patient care.
Data exclusions	Exclusion criteria were pre-established. We excluded patients younger than 18 years old at the time of ICU admission, patients where mortality was not documented and patients with evidence of withdrawal of treatment. In MIMIC-III, we excluded patients where positive end-expiratory pressure (PEEP), fraction of inspired oxygen (FiO2) and ideal body weight- adjusted tidal volume (Vt) is missing or in case of in sufficient data. In eICU-RI, we excluded ICU readmissions and patient admitted in an ICU with insuffícent data collection.
Replication	The results were reliably reproduced in a large array of sensitivity analyses, as described in Methods and Extended Data.
Randomization	Not relevant, this was not a randomized controlled study.
Blinding	Not relevant, this was not a randomized controlled study.

Reporting for specific materials, systems and methods

We require information from authors about some types of materials, experimental systems and methods used in many studies. Here, indicate whether each material, system or method listed is relevant to your study. If you are not sure if a list item applies to your research, read the appropriate section before selecting a response.

Materials & experimental systems

n/a	Involved in the study	
☒	☐	Antibodies
☒	☐	Eukaryotic cell lines
☒	☐	Palaeontology and archaeology
☒	☐	Animals and other organisms
☐	☒	Human research participants
☒	☐	Clinical data
☒	☐	Dual use research of concern

Methods

n/a	Involved in the study	
☒	☐	ChIP-seq
☒	☐	Flow cytometry
☒	☐	MRI-based neuroimaging

Human research participants

Policy information about studies involving human research participants

Population characteristics	The research involved a total of 96,156 unique adult patients admitted to 133 separate intensive care units in the USA. The average age was 65 years old and 53% of the subjects were male. All the patients were mechanically ventilated.
Recruitment	There was no recruitment. Only retrsopective data from MIMIC-III database and eICU database
Ethics oversight	Approval of data collection, processing, and release for the MIMIC-III database has been granted by the Institutional Review Boards of Beth Israel Deaconess Medical Center (Boston, MA, USA) and the Massachusetts Institute of Technology (Cambridge, MA, USA). Approval of data collection, processing and release for the eICU database has been granted by the eICU research committee and exempt from Institutional Review Board approval. All data was processed on the computational infrastructure of the Rheinisch Westfälische Technische Hochschule (RWTH) Aachen University and the University Hospital RWTH Aachen in accordance to European Union data protection laws.

Note that full information on the approval of the study protocol must also be provided in the manuscript.
